# Rethinking success, integrity, and culture in research (part 2) — a multi-actor qualitative study on problems of science

**DOI:** 10.1186/s41073-020-00105-z

**Published:** 2021-01-14

**Authors:** Noémie Aubert Bonn, Wim Pinxten

**Affiliations:** Research Group of Healthcare and Ethics, Faculty of Medicine and Life Sciences, Hasselt University, Martelarenlaan 42, 3500 Hasselt, Belgium

**Keywords:** Research integrity, Research culture, Research assessment, Pressure to publish, Inter-actor dialogue, Success in science, Misconduct, Questionable research practices, Flanders, Research evaluation

## Abstract

**Background:**

Research misconduct and questionable research practices have been the subject of increasing attention in the past few years. But despite the rich body of research available, few empirical works also include the perspectives of non-researcher stakeholders.

**Methods:**

We conducted semi-structured interviews and focus groups with policy makers, funders, institution leaders, editors or publishers, research integrity office members, research integrity community members, laboratory technicians, researchers, research students, and former-researchers who changed career to inquire on the topics of success, integrity, and responsibilities in science. We used the Flemish biomedical landscape as a baseline to be able to grasp the views of interacting and complementary actors in a system setting.

**Results:**

Given the breadth of our results, we divided our findings in a two-paper series with the current paper focusing on the problems that affect the integrity and research culture. We first found that different actors have different perspectives on the problems that affect the integrity and culture of research. Problems were either linked to personalities and attitudes, or to the climates in which researchers operate. Elements that were described as essential for success (in the associate paper) were often thought to accentuate the problems of research climates by disrupting research culture and research integrity. Even though all participants agreed that current research climates need to be addressed, participants generally did not feel responsible nor capable of initiating change. Instead, respondents revealed a circle of blame and mistrust between actor groups.

**Conclusions:**

Our findings resonate with recent debates, and extrapolate a few action points which might help advance the discussion. First, the research integrity debate must revisit and tackle the way in which researchers are assessed. Second, approaches to promote better science need to address the impact that research climates have on research integrity and research culture rather than to capitalize on individual researchers’ compliance. Finally, inter-actor dialogues and shared decision making must be given priority to ensure that the perspectives of the full research system are captured. Understanding the relations and interdependency between these perspectives is key to be able to address the problems of science.

**Study registration:**

https://osf.io/33v3m

**Supplementary Information:**

The online version contains supplementary material available at 10.1186/s41073-020-00105-z.

## Background

When performing scientific research, researchers agree to abide by principles and standards of practice. We know, however, that best practices are not always upheld [1–3] {Pupovac, 2014 #750;Martinson, 2005 #753;Fanelli, 2009 #2908}. Obvious deviations from accepted practices are generally known as misconduct. But misconduct is difficult to define. At the moment, one of the most widely accepted definitions of misconduct comes from the US Department of Health and Human Services 42 CFR Part 93. This definition is endorsed by the US National Institute of Health (NIH) and Research Integrity Office, and defines misconduct as “fabrication, falsification, or plagiarism in proposing, performing, or reviewing research, or in reporting research results”. Nonetheless, the definition also specifies that “research misconduct does NOT include honest error or differences of opinion” [4]. In other words, even in its simplest definition, misconduct remains contextual and nuanced, further complicating what constitutes research integrity. Adding to this complexity, several behaviours which cannot be characterised as manifest misconduct are also thought to deviate from research integrity. These behaviours, referred to as questionable — or detrimental — research practices, are so common in the scientific community [2, 3] that their cumulative damage is believed to surpass the damage of manifest misconduct [5]. Nonetheless, questionable research practices are not univocally condemned, adding to the challenge of distinguishing acceptable from unacceptable practices.

Beyond the complexity of identifying which behaviours transgress research integrity, the causes that may lead to integrity deviations also bring confusion and disagreement. A vast body of research on the topic suggests that both personal and environmental factors are at play. Some studies condemn personal factors such as ego and personality (e.g., [6–13]), gender (e.g., [14, 15]), and career stage (e.g., [2, 16]). A few others instead believe that researchers’ lack of awareness of good practices (e.g., [17–19]), inadequate leadership modelling and mentoring [20, 21], and inefficient oversight [22] are to blame. But some studies also suggest that issues embedded in the research system are at play [23]. Among those, the pressure to publish (e.g., [24–28]), perverse incentives and conflicting interests (e.g., [29–32]), and competition [28] are the most frequent suspects. In light of these works, integrity seems to depend on a complex interactions between individual factors, lack of awareness, and research climates.

Despite the rich body of research available to explain what threatens research integrity, few empirical works target the perspectives of the stakeholders beyond researchers [33]. Given the diversity of actors involved in research systems, focalising the integrity discourse on researchers inevitably overlooks essential voices.

To add some of the forgotten voices to the discourse and understand how non-researchers perceive scientific climates, we captured the perspectives of policy makers, funders, institution leaders, editors or publishers, research integrity office members, research integrity community members, lab technicians, researchers, research students, and former-researchers who changed career on the topics of success, integrity, and responsibilities in science. We used the Flemish biomedical landscape as a baseline to be able to grasp the views of interacting and complementary actors. We present our results in two publications. In the present paper, we present the problems that were identified as affecting the integrity and the culture of science, and in an associated publication [34] we discuss how different actors perceive success in science.

## Methods

For reader’s convenience, the methods of our project are described in full both here and in our associated paper [34].

### Participants

The present paper reports findings from a series of qualitative interviews and focus groups we conducted with different research actors. This qualitative work was part of the broader project Re-SInC (Rethinking Success, Integrity, and Culture in science; the initial workplan is available at our preregistration [35]).

In Re-SInC, we captured the views of different research actors on scientific success, problems in science, and responsibilities for integrity. Being aware that the term ‘research actor’ may be ambiguous, we defined research actors as any person having a role in the setup, funding, execution, organisation, evaluation, and/or publication of research. In other words, we included actors linked to the policing, the funding, the evaluation, the regulation, the publishing, the production (i.e., undertaking the research itself), and the practical work of research, but we did not include sole consumers of science or end users of new technologies.

We used Flanders as a setting, including participants who either participate in, influence, or reflect (directly or indirectly) upon the Flemish research scene. We will discuss below that our findings are also highly coherent with similar works in different research settings (see for example [21] in Croatia, [23] in Denmark, [28] in the US, and [36–38] in the UK). In most cases (49 out of 56 participants), participants did not know the interviewer before the interviews and focus groups. In selecting participants, we aimed to capture the breadth of the Flemish research scene. Using Flanders as a research setting had the advantage of allowing us to capture perspectives from an entire research system in a feasible setting. The Flemish research scene comprises of five main universities and a number of external research institutes, major funding agencies, a federal research policy department, and one advisory integrity office external to research institutions. We chose to concentrate our research on three of the five universities, and to include partnering European funding and policy organisations as well as international journals and publisher to build a realistic system sample. When participants were affiliated with a university, we focused on the faculty of biomedical sciences. Given the exploratory and qualitative nature of this project, we did not aim for an exhaustive nor a fully representative sample. Our objective was to shift the focus from the narrow view targeting mainly researchers to a broader view that includes a broad range of research actors. Accordingly, we maximized the diversity of participants in each actor group to ensure that each group encompassed a wide range of potentially different perspectives.

Our main actor categories are PhD students, post-doctoral researchers (PostDoc), faculty researchers (Researchers), laboratory technicians (LT), policy makers and influencer (PMI), funding agencies (FA), research institution leaders (RIL), research integrity office members (RIO), editors and publishers (EP), research integrity network members (RIN), and researchers who changed career (RCC). The composition of each actor group is detailed in Table 1.

**Table 1:** Demographics of participants

Actor group	Abbrev.	Sample description	N, setting, and gender^a^
Researchers	Researchers	Faculty researchers from the Faculty of Medicine and Life Sciences of the host institution.	[**■ ■ ▲ ▲**]
Post - doctoral researchers	PostDoc	Post-Doctoral researchers from the Faculty of Medicine and Life Sciences of the host institution.	[**■ ■ ■ ▲ ▲**]
PhD students	PhD	PhD students enrolled in the Faculty of Medicine and Life Sciences of the host institution.	[**■ ■ ■ ■ ■ ■**]
Lab technicians	LT	Laboratory technicians from the Faculty of Medicine and Life Sciences of the host institution.	[**■ ■ ■ ■ ■**]
Past researchers who changed career	RCC	Although this group was not part of our pre-registration, one RCC asked us whether she could take part in our study after seeing the invitation email. After having a chat with her, we realized that hearing the narrative and perspectives of individuals who did research work but decided to leave academia would deeply enrich our results and inform us on problems which are big enough to drive researchers away from research. Therefore, we invited a few researchers who changed careers (i.e., researchers or research students who decided to leave academia) to participate in interviews. In this group, we selected individuals from each of the three universities included in our project, and ensured to have individuals who left academia during their PhD, after their PhD, after their PostDoc, and during a tenure track. Recruitment of those participants was helped by recommendations from colleagues who were aware of the profiles we were looking for.	**■ ■ ■ ■ ▲**
Research institution leaders	RIL	We included three Flemish universities in our study. In each institution, we involved several members from the board of directors. These included directors of research, deans, or directors of doctoral schools from the faculties of medicine and life sciences or equivalent.	**▲ ▲ ▲ ▲ ▲ ▲ ▲**
Research integrity office members	RIO	We included different members from offices in charge of investigating allegations of research integrity and misconduct in three Flemish research institutions and outside research institutions in Flanders (e.g., research integrity officer, policy officers, etc.)	**■ ■ ■ ▲**
Editors and publishers	EP	We invited both big and small editors and publishers, and were fortunate to be able to involve journals and publishers with a broad range of editorial practices (i.e., open access and subscription based; published in local language and published in English; focusing on reviews and focusing on ground breaking empirical findings). To select the interviewees, we first invited a selection of journals from the top twenty highest Impact Factor for 2017 under the category of ‘Medicine, general and internal’ in the Journal Citation Reports (Clarivate Analytics), purposively picking different publishing models. In addition, we invited select publishers to take part in our research. After conducting individual interviews with a few agreeing participants from this sub-selection, we organized a small focus group with editors of smaller or differing journals, allowing us to involve a great diversity of editors and publishers.	[**■ ■ ▲ ▲**] **■ ■ ■ ▲**
Funding agencies	FA	We selected national, as well as European funding agencies, making sure to target different funding styles. We made sure to include perspectives from regional public funders, regional private funders, international funders, as well as funders focusing on applied research and funders focusing on fundamental research.	[**▲ ▲] ■ ▲ ▲**
Policy makers or influencers	PMI	In this group, we included both organisations responsible for setting science policy, and organizations which influenced such policies by serving as informers. Consequently, PMIs do not necessarily write nor decide science policies, but may also be asked to provide data which later influences policy decisions.	**■ ▲ ▲ ⬤**
Research integrity network members	RIN	We selected a few actors from the research integrity core experts. These included researchers involved with important European research projects on research integrity as well as one actor involved in writing the European Code of Conduct for Researchers.	**■ ▲ ▲**
TOTAL = 56 participants			

^a^Square bullets **(■)** represent female participants; triangle bullets (▲) represent male participants, and round bullets (⬤) represent participants with undefined gender (‘prefer not to answer’). Bullets displayed in brackets represent participants with whom we conducted as focus groups or joint interviews

It is important to keep in mind that the research world is complex and not organized in distinct actor groups. Consequently, participants could often fit in more than one category, and sometimes felt the need to justify circumstances that would make them fit in the category we selected. Before the interview, we asked participants whether they agreed with the category we assigned them in, and we refined and exemplified the definitions of our actor groups to reflect the participants’ distinctions (i.e., further explaining the slight differences between the groups planned in the registration and those used here).

### Recruitment

We used several recruitment strategies. For the focus groups with PhD students and researchers, we circulated an email to everyone in the Faculty of Medicine and Life Sciences of the host university and invited them to register on an interest list. We later scheduled a convenient time where most of those who registered were available. We used a similar strategy for the focus group of editors and publishers, but circulated the invitation in a relevant conference. For focus groups with lab technicians and post-doctoral researchers, key players helped us recruit and organize the focus group. Estimating response rates for the focus groups would thus be challenging.

For interviews, we invited participants directly via email. We sent up to three reminder emails, but did not pursue further if no response was obtained at the third reminder email. All participation was on a voluntary basis. From the individuals invited to participate in an interview, two declined the invitation, one did not feel like a good fit for our project and was therefore not interviewed, and eight did not reply to our invitation. Although invited individuals sometimes referred us to colleagues holding similar roles in the same institution, the remaining invitations led to interviews.

### Design and setting

We conducted semi-structured interviews and focus groups, meaning that we asked broad questions in an open manner to target main themes rather than specific answers. All interviews and focus groups were audio recorded and transcribed verbatim. Details about the tools used to guide the interviews and focus groups are available in the tool description below.

To maximise transparency, we provide an extended descriptions of the interviewer and the setting of the interviews in Supplementary file section 1 and a copy of the COnsolidated criteria for REporting Qualitative research checklist (COREQ) in Supplementary file section 2.

### Ethics and confidentiality

The project was approved by the Medical Ethics Committee of the Faculty of Medicine and Life Sciences of Hasselt University (protocol number CME2016/679), and all participants provided written consent for participation, for use and publication of anonymized direct quotes, and for dissemination of the findings from this project. A copy of the consent forms is available in the registration of this project [35]. We protected the confidentiality of participants by removing identifiers from quotes included in the text. Nonetheless, Flanders is a small research system and given our actor-specific sample, personal identification within quotes remains a risk despite our efforts. To further protect participants’ confidentiality and avoid that identification of individual quotes lead to identification of all quotes from the same participant, we decided not to specify respondents in individual quotes, but to refer only to actor groups.

Following this reasoning, we are unable to share full transcripts, but attempted to be as transparent as possible by providing numerous quotes in the text, in tables, and in the supplementary file.

### Tool

To build our focus group guide, we inspired our style and questions from the focus group guide developed by Raymond De Vries, Melissa S. Anderson, and Brian C. Martinson and used in a study funded by the NIH [28]. We obtained a copy of the guide after approval from the original authors, and revised the guide to tailor questions to the topics we wished to target, namely ‘success in science’ and ‘responsibilities for research integrity’. We revised our focus group guide several times before data collection and discussed it with Raymond De Vries — expert in qualitative inquiries and part of the team that built the original guide upon which we inspired ours. We built interview guides based on our revised focus group guide. We adapted specific questions (e.g., responsibilities, evaluation) to each actor group, but preserved the general structure and themes for all interviewees. A general version of the interview and focus group guides are available in Supplementary file parts 3 and 4. More specific group guides can be provided upon request. All guides were constructed around the following four topics:

i) **Success in science**: What makes a researcher successful? Are these characteristics captured in current assessments? What are indicators for success? What do you feel is most satisfying, most rewarding about your career (specifically for researchers and research students)?
ii) **Current problems** (including misconduct and questionable research practices): Do you have experience with research that crossed the lines of good science? How can we draw the line, what are red flags? Why do bad practices happen? Can they happen to anyone?
iii) **Responsibilities towards integrity**: What is your responsibility towards integrity? Where does it end? Who else is responsible? In what ways are other actors responsible?
iv) If you were granted a fairy wish and could **change one thing in how science works**, what would you pick?

It is important to consider that the interview guide was not used mechanically like a fixed questionnaire, but sometimes shortened, expanded, or reordered to capture responses, interest, and to respect time constraints. In this manuscript, we mainly report findings from the second (ii) and third (iii) topics, although we sometimes captured the discussion from other questions when relevant.

### Analysis

Recordings were first transcribed verbatim and, where necessary, personal or highly identifiable information was anonymized. We analyzed the transcripts using an inductive thematic analysis with the help of NVivo 12 Software to manage the data. The analysis proceeded in the following order:

i) **Initial inductive coding**: NAB first analyzed two focus groups (i.e., researchers and PhD students) and five interviews (i.e., RIL, RIO, PMI, RCC, and RIN) to have an initial structure of the themes targeted. In this step, she used an inductive thematic analysis [39] while keeping the three main categories — i.e., success, integrity, and responsibilities — as a baseline. Using the inductive method avoided that we limit our analysis to the order and specific questions included in our guide, and allowed us to also identify and note themes that were raised spontaneously or beyond our initial focus.
v) **Axial coding**: With this first structure, NAB and WP met and took a joint outlook at these initial themes to reorganize them in broader categories and identify relationships between categories. For this step, NAB built figures representing connections between the main themes, and refined the figures and the codes after the meeting.
vi) **Continued semi-inductive coding**: NAB continued the coding for the remaining transcripts, sometimes coding deductively from the themes already defined in steps 1 and 2, and sometimes inductively adding or refining themes that were missing or imprecise.
vii) **Constant comparison process**: NAB and WP repeated the axial coding and refining steps several times throughout this process, constantly revisiting nodes (i.e., individually coded themes) by re-reading quotes. The nodes and structure were then discussed with RDV to reconsider the general organisations of the nodes. This constant comparison process is common in qualitative analyses, and is commonly used, for example, in the Qualitative Analysis Guide of Leuven (QUAGOL [40];). This repeated comparison led to a substantially solid set of nodes which later guided further coding in a more deductive manner, though we made efforts to remain open to possible new themes in respect of our inductive analysis.
viii) **Lexical optimization**: Finally, after having coded all transcripts, NAB and WP further discussed the choice of words for each node and reorganized the themes to ensure that they were an ideal fit for the data they were describing. NAB and Raymond De Vries met to have a final outlook of the general structure, and to reorganise the nodes in clean and natural categories.

## Results

### Short summary of results

Fifty-six participants spread in eleven different actor groups took part in our interviews and focus group discussions. From their responses, participants revealed conflicting views on the problems that affect the culture of science and threaten integrity, both between and within actor groups.

We noticed that the jargon which is normally used to discuss misconduct and integrity was not common to all research actors. The core reasons for taking integrity issues seriously also seemed to differ between individuals and actor groups, ranging from the personal intentions to the negative impact on scientific knowledge. Most respondents identified excessive pressures as likely causes of integrity issues, yet many considered that researchers were still ultimately responsible for their actions.

Interviewees seemed more inclined to discuss the general problems which may deter research integrity than to discuss instances of misconduct and questionable research practices. From the problems described, we identified two general categories of issues: Issues related to (i) personalities and attitudes, and (ii) research climates. Issues related to personalities and attitudes were mentioned as potential targets for employers to consider, but were also admitted to be rather immutable. Issues linked to the research climates highlighted problems which resulted from existing research environments and research culture. Among those, issues in mentorship and support of researchers were thought to impact researchers’ understanding of good scientific practices. Structural issues, such as the precariousness and scarcity of research careers, especially problematic for young researchers, were thought to be a major issue which aggravated the threatening impact of pressures and perverse incentives. Overspecialisation, expectations of versatility, and the lack of collaboration also came into play as constraining the time available for research, further intensifying the pressures on researchers and reducing the possibility for control and monitoring. Deeper in the culture attached to research, the care and support given to researchers was also noted as being limited. Expectations that researchers should devote themselves to science were said to impact the well-being and satisfaction of researchers. A general culture of profit, intolerance for failure, and expectations of extraordinary results added up to fuel a culture of ‘publish-or-perish’. Growing competitiveness and lingering hierarchies were believed to influence how research was planned, performed, and reported.

Finally, when asked about responsibilities for change, interviewees revealed a shared feeling of helplessness towards current problems. They felt that issues were caused by inadequate decisions of different actors, and thus felt frustrated and lost their trust in other actor groups.

Complete and detailed results are presented in the subsections below.

### Integrity jargon

Although it was not our explicit objective to analyze the familiarity of respondents with jargon, we noticed that many respondents were not familiar with key terms such as falsification, fabrication, plagiarism (FFP), misconduct, and questionable research practices (QRP). While research integrity office members, research integrity network members, and editors or publishers did refer to such key terms, many interviewees including funders, policy makers or influencers, researchers, and some institutions leaders appeared less familiar with this jargon. They would use descriptions such as ‘changing your data’, ‘faking data’ and ‘cheating’, rather than the more familiar FFP and QRP terms. Even the term ‘misconduct’ was rarely used, most often replaced by ‘fraud’.

The unfamiliarity with integrity jargon may partly be due to Dutch-speaking nuances or to our sampling strategy (i.e., we intentionally included interviewees who were not integrity experts in order to obtain a perspective that was unbiased by the integrity literature). Nonetheless, this finding also means that researchers working on research integrity should be aware that common terms such as FFP, misconduct, QRP, and other key terms may still be jargon to actors who are, ultimately, the intended audience.

### Integrity issues

As we discussed in the section on jargon, respondents were not consistent in their use of terms to refer to problems in sciences. Some spoke for example about ‘bad research practices’, others about ‘problems in science’, ‘science that is not in line with what it should be’, ‘suboptimal science’, ‘integrity problems’, or ‘misconduct’. We granted respondents the liberty to formulate their thoughts as they preferred, regardless of jargon, to maximally capture their views and opinions. Given this lack of consistent terminology, we will further refer to ‘integrity issues’ for all response obtained when probing for what constitutes integrity problems such as misconduct and questionable research practices.

Many interviewees started by explaining that integrity issues were heterogeneous, ranging on a broad continuum between questionable research practices and manifest misconduct. While some issues are manifestly incompatible with good scientific practice, the (un)acceptability of others might be less clear. This continuum raised doubts about what can be tolerated and what should be sanctioned. Manifest misconduct was perceived as the exception rather than the rule, and the more common questionable research practices were thought to be a more important problem.

*“The experience that I have in research is that really [misconduct] is exceptional. It makes … It’s breaking news, because it’s something that we, in the community of research, we consider inacceptable, but it’s rare. It’s rare.” (FA)*

*“The very serious misconduct is not such a big problem. It’s … it’s more the grey area that is a problem because of, yeah, the amount of … of, yeah, the bad practices.” (RIO)*

Interviewees also explained that perspectives of integrity issues evolved over time, challenging accusations of past misbehaviours. For most interviewees, the biggest challenge in detecting integrity issues was the difficulty to prove intention, even in the case of manifest misconduct. Interviewees who had had to deal with misconduct cases mentioned being convinced of intentionality, but often missing the elements to prove it.

*“But I know that there is a problem with integrity in that person. I can feel it. We have no proof.” (RIL)*

*“We had this case once of a guy … and I, up until now, I'm still convinced that he completely fabricated his research. I know for sure that he did. But we weren't able to prove it because it's very difficult to prove that something is not there. [ … goes on to describe the case in which the researcher deleted all possible evidence ...] So there was no proof anywhere. And it was the adding up of all these coincidental things that made us believe – and in fact of course his attitude and the entire person, him as a person being was very unreliable with a lot of lies, with a lot of contradictions, stories that didn't add up, very negatively threatening … so it was a very nasty one. And at that point you sense that there is something off.” (RIO)*

**Fig. 1:**
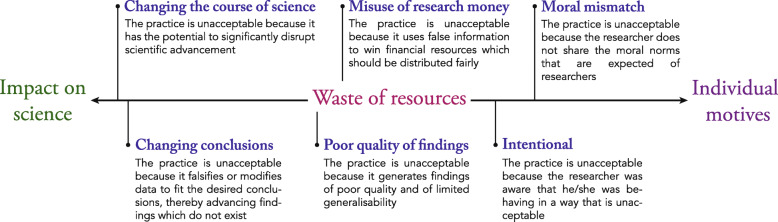
What makes questionable practices unacceptable?

We found that the reason for taking integrity issues seriously ranged from general worries about the impact on science to worries about the morality and motives of researchers. We depicted these main perspectives in Fig. 1, and illustrated each perspective with quotes in Supplementary file part 5.

Respondents’ perspectives were mixed and diverse, but some group-specific characteristics could be observed. Among those who worried most about the impact on science, the potential to alter conclusions or change the course of science was what made integrity issues troublesome. Editors and publishers were particularly strong on this view. Although they acknowledged the importance of intention in identifying and sanctioning misconduct, editors and publishers emphasized that, given their late entry in the research process, their main concern was the effect that misconduct may have on the scientific record. Some institution leaders also shared this perspective but added that conscious decisions could also alter the seriousness of integrity issues. For example, one interviewee mentioned that integrity issues caused by laziness might be of a different order than intentional fabrication or falsification for personal benefit.

*“The only misconduct I’ve picked up was just stupidity. PhD students who scanned a little too short and had to go back to the scanner and thought “I could just copy-paste the bottom bit because there’s nothing on it anyway“. That’s real misconduct, but at the same time, that’s not scientific fraud. Well it was, it is scientific fraud, but he was not changing a conclusion, he was just too lazy to scan a really nice experiment [...] What I consider cheating is that you leave out the data that don’t suit your model. Or you make up data to get your model correctly. That is what I call cheating.” (RIL).*

Although both cases are the result of conscious decisions, in the first case science is harmed as a side effect of pursuing a goal extrinsic to science (i.e. laziness), while in the second case science is harmed by explicitly by going against its intrinsic goals (i.e. leaving out data to suit one’s model).

The same interviewee later mentioned that small fraudulent publications in early stages of research might not be so problematic since they were likely to be corrected early on and had little risk of changing the course of science (See quotes in Supplementary file section 5). One policy maker proposed that producing weak or low quality results which could not be generalized or used for further research was also “*sloppy or bad practice*” since the results will not represent reality. One research funder supported this perspective by adding that poor quality research and delays in delivery were crucial to them since their goal was to “*guarantee the most efficient use of public money*”. Somewhere between these views, other interviewees argued that misuse of research money immediately constituted misconduct and that any misrepresentation or duplication in an application done purposively “*to win money”* should be considered fraud. Finally other interviewees focused on individual intentions rather than on the impact on research and resources. Research integrity office members were most represented among those who believed that intention and morals were the most important aspect to determine misconduct. One interviewee explicitly mentioned that even if the conclusions were unchanged and the results were simply slightly embellished, the intention and moral mismatch was what made such practices inacceptable.

*“[Sometimes researchers say] 'yeah but it didn't change the main results of my article, so what's the problem?' [...] OK if the results are being the same, that's not the issue actually, it should be the process also. And at that point you see that there is this moral mismatch.” (RIO)*

#### Can integrity issues happen to anyone?

When asking respondents whether integrity issues could happen to anyone, the answers were varied and contradicting. Many respondents supported that certain types of personalities were more prone to integrity issues than others. The extent of this perception, however, varied from interviewee to interviewee, and appeared to be linked to personal experiences with misconduct cases rather than to actor groups.

First, some interviewees perceived researchers as inherently good by default. Statements along the lines of “*I believe in the goodness of researchers*” (RIO), “*She was a real scientist … I could not believe that she would ever, yeah, <commit> misconduct on purpose*.” (RCC), or “*I find it very hard to believe that somebody who would go into science, go into research to intend really to go and do wrong things.*” (RIO) illustrate this perspective. Nonetheless, the same interviewees later explained that despite researchers’ inherent goodness, academia sometimes placed so much pressure on researchers that it may push them to compromise integrity. Adding to this view, other interviewees proposed that certain researchers were more prone to misconduct than others.

*“I definitely think there is a pathological end of the spectrum.[ … ] But I also think that there is so much pressure, especially on people at the beginning of their careers, that I don’t think anyone is completely immune to actually committing something.” (EP)*

*“The truly white and the truly blacks are rare. [ … ] many people will be willing to cut a small corner somewhere in an experiment. But really cutting a corner meaning 'I come up with an answer that I don't have yet, but I assume it will be this and I'll give myself the data for free'... I think requires a mentality.” (RIL)*

A minority of interviewees even believed that individual characteristics were the biggest (if not sole) determinant of integrity. Although this perspective was only supported by a few interviewees, supporters of this view questioned the benefit of training and support in promoting research integrity, and rather asserted that, to build good researchers, institutions must choose the right individuals.

*“Sloppy science, first and foremost is the product of sloppy scientists. It’s not the product of a system, it’s the product of a person. [ … ] there are persons who are striving for high levels of integrity, and there are people who are not doing so.” (PMI)*

*“Integrity is in the person. [ … ] integrity is something that is in you. You have it or you don't have it. I mean you have it, it’s there. And when you don’t have it, you don’t have it. So we cannot create integrity, it's something that's in the people. Working together and being involved, that’s something [universities] can create by offering a structure. But I'm a strong believer that the integrity is inborn, it's in you.” (RIL)*

Consequently, respondents generally conceded that no one is immune to integrity issues when pressures and contributing environmental factors are excessive. Nonetheless, most respondents also supported that certain individuals were more prone to integrity issues than others, in particular in the case of serious misconduct.

### The problems of science

**Fig. 2:**
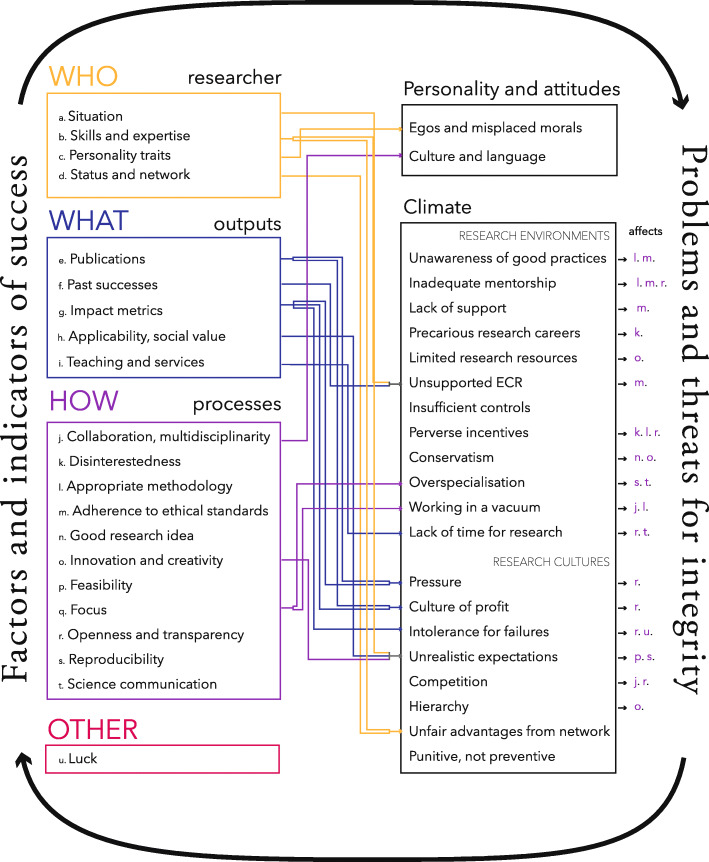
Although this figure oversimplifies the complex interaction between success and integrity, it shows how diverse and circular the connection is, with both success generating problems, and problems influencing and blocking the processes needed for success. ECR stands for early career

In performing our interviews, we noticed that what respondents were most concerned with was not integrity issues per-se, but rather a number of more general problems that may affect the integrity and the research culture. We organized the different problems mentioned in two big categories: problems related to the *personality and attitudes of individual researchers*, and problems related to the *research climates* in which we identified issues the organizational structures imposed on researchers as well as the research culture that surrounds them. In Fig. 2, we sketch the connection between the themes of success that were raised in the first part of this study [34] and the different problems of science raised below. Despite the oversimplification of this illustration, we can see at first glance that the two topics are highly interconnected. In the following sections, we detail the different problems of science that were raised by interviewees and explain their impact on research integrity and research culture. We complement our thematic findings with an extensive selection of relevant quotes available in Supplementary file section 6.

#### Issues related to the personality and attitudes

As we described above, interviewees mentioned several individual characteristics that could be problematic and might impact the integrity of research. Personal egos and misplaced morals, or the ‘bad apple’ idea, was recurrently mentioned as a possible explanation for misconduct. The high prevalence of interviewees mentioning these aspects might have been primed by our question ‘can misconduct happen to anyone?’, yet many interviewees spontaneously mentioned the influence of personalities and morals on misconduct. Respondents especially linked personal characteristics to ‘big misconduct cases’ such as the cases that appear in the news. A few personal characteristics mentioned also raised conflicting dualities with what was believed essential to success. For instance, interviewees supported that ambition, passion, and tenacity were key elements of success (see [34] for a detailed description of success), while also arguing that hyper-ambition or excessive desire to be successful could bias conclusions and encourage researchers to loosen their integrity. Several respondents also associated problematic attitudes with cultural backgrounds proposing, for instance, that foreign students sometimes perceived the seriousness of detrimental research practices differently. Cultural and language differences were also mentioned as challenging the ability to communicate and as increasing the risk of loneliness, misunderstandings, and mistrust that may lead toward concealment. Although few approaches were offered to counter personal threats to integrity, some interviewees proposed that the social activities put in place within a university (e.g., office parties, lunch break, etc.), may help to build a welcoming environment where communication is facilitated. Nonetheless, interviewees also explained that past attempts at creating a more integrative environment had suffered from language barriers; Belgian researchers and students would generally speak in Dutch during lunch break and activities, keeping a divide between them and international PhD students.

#### Issues related to the research climates

##### Lack of knowledge of good practices

Several respondents mentioned that researchers were sometimes unaware of good practices. Lack of knowledge of good practices was not only perceived as a problem of individual researchers who lack insight in their own behaviour, but also as a systemic issue caused by insufficient training and inadequate mentoring within the larger scientific community.

##### Insufficient support, mentorship, and guidance

Problems from the lack of mentorship and guidance for researchers were also very preeminent in our responses. This issue was discussed on different levels. One the one hand, students mentioned that they lacked guidance, support, and time from their supervisors. PhD students and researchers who changed career were especially vocal on this point. Although the lack of mentoring in such cases is not necessarily causing an unfamiliarity with good practices, young researchers often felt lonely, stressed, and frustrated about the lack of support they receive. One research integrity community member explained that loneliness was often a red flag for integrity issues. On the other hand, researchers themselves mentioned that they lacked support and guidance on how they should meet integrity and ethical standards. For example, one researcher mentioned that funders tended to increase the number of “*tick boxes*” without increasing training and capacity. Along the same line, one participant in the RIO group observed that integrity training generally comes in the form of specialized, intensive courses, when in fact it should be integrally embedded throughout the research training in order to become “*part of the research process*” for every researcher.

##### Precariousness of research careers and lack of support for early career researchers

The precariousness of research careers and the constant insecurity linked to short-term contracts and scarce opportunities for advancement was another recurrent issue mentioned by our interviewees. One policy maker or influencer explained that, in Flanders, the number of students completing a PhD highly exceeds the number of academic positions available, and that despite this imbalance, the current number of PhD students in Flanders was still below the target set by the Organisation for Economic Co-operation and Development (OECD). As a result, most PhD students will have to find a career outside academia, often finding careers at a Masters level rather than at a PhD level. For early career scientists (including participants from the groups of PhD students, PostDoc, Researchers, and RCC) the insecurity from uncertain career opportunities was evident and was thought to be accentuated by the steep hierarchies that dominate academia. One researcher who left academia recalled stories of supervisors who intimidated PhD students by telling them that they could be replaced at any time if they didn’t meet expectations. This lack of employment security, in turn, could risk compromising the honesty and openness of students who fear losing their job if they commit a mistake or fail an experiment. Adding to the lack of stability and security embedded in research careers, young researchers also felt unsupported while juggling with too many tasks to be able to focus on the outputs required for advancing their career. Given that grant attribution often depends on past achievements, early career researchers feared that they were disadvantaged and often left out from the competition, which ultimately only advantaged “*big names*”. Adding to the employment insecurity, excessive pressures for output, insufficient resources, and inability to compete with established researchers, early career researchers often faced life responsibilities which required a need for stability and assurance of continued employment (i.e., building a family, buying a house, caring for aging parents, etc.). Indeed, most interviewees who left academia motivated their decision to leave by the desire for a stable career with a sane work–life balance. Two of those interviewees recalled clear symptoms of burnout, and all recalled a certain distress from their time in academia. Yet, leaving academia also came with an important emotional burden. Even though all former-researchers interviewed expressed a sense of relief from leaving academia, most admitted that the decision to leave had been difficult to make. Some even perceived that they left academia because of their inability to be “*real researchers*” rather than because of the system’s unreasonable demands (see relevant quotes in Supplementary file part 6). The current system can thus impose important disappointments, self-doubt, and emotional distress on early career researchers for whom future employment is uncertain.

##### Inefficient controls and perverse incentives

Inefficient means to detect integrity issues, added to inadequate incentives were also thought to be a problem of current research environments. Given the high level of specialisation of research activities, interviewees worried that misconduct and detrimental practices often go unnoticed or unsanctioned. In addition, not only is there a lack of incentives for practices that ensure the quality and integrity of research, but the incentives in place were said to encourage dubious research practice. For instance, the expectation that researchers should come up with “*big bold claims*” and “*ground-breaking*” or “*extraordinary*” results despite working with short-term funding schemes were said to encourage research on smaller populations and to incite inappropriate statistical controls and analyses to inflate significance.

##### Conservatism

Adding to the above concerns, funding distribution was also criticized for being conservative and for discouraging high risk research (i.e., research with important possible outcomes but with high potential for yielding negative results). As a coping strategy, both PhD students and researchers admitted having heard of situations where applicants obtain funding for projects that have already been conducted in order to use the money for truly innovative research that would otherwise be difficult to fund. Nevertheless, researchers considered such strategies to be problematic since they infringed the funding agreement while reinforcing the conservatism of research funding schemes. In response to these concerns, one research funder proposed that smaller, private funders “*could, and therefore also probably should <be> somewhat more risk taking than public funders.*”

##### Overspecialisation, working in a vacuum, and lack of time for research

Interviewees also shed light on a problematic interplay between overspecialisation, isolation, and lack of time for research. Overspecialization was criticized for potentially deterring the replicability of research, thereby undermining the detection of mistakes and misconduct. But overspecialisation was also criticized for increasingly isolating researchers from one another and discouraging collaborations. Interviewees often felt that researchers work in a vacuum rather than within a shared community. PhD students admitted that research was “*sometimes a bit lonely*”, and that their supervisors even discouraged external collaborations, public presentations, or data sharing by fear of being scooped or of diluting the recognition for their work (see relevant quotes in Supplementary file section 6). But even within universities, many conceded that researchers often “*don’t even know what is happening within their own buildings*” and that this isolation probably leads to unnecessary duplication and waste of research resources. Working alone also means that researchers are expected to have highly versatile skills to be able to fulfil all the expectations of their position. The three main pillars — research, education, and services — were further criticized for being inflexible and for asking researchers to conform to a one-size-fits-all model that disregarded personal skills and discouraged team efforts. With the added bureaucratic burdens and the “*tremendous amount of meetings*” reported by researchers, many felt that this lack of time for research fed into bigger problems by decreasing the quality of mentorship, research, and education.

##### Pressure

Deeper into the habits and customs of researchers, several issues embedded in the culture of research were also seen as problematic. The pressure to perform — and especially to deliver — and the culture of publish or perish were the issues that were mentioned by the biggest number of interviewees. Interviewees described that pressures threatened the quality of science, impeded on researchers’ health and happiness, and could potentially lead to misconduct. By listening to multiple research actors, however, we discovered that pressures are multilevel and that they affect more than researchers alone. For instance, institution leaders felt for their institute a pressure to deliver more and faster in order to sustain excellence and increase their attractiveness on the international scene. Interviewees explained that, in systems where structural funding depends largely on institutional outputs such as publications and impact factors,^1^ institutions must continuously increase their outputs to keep their share of structural funding. As a result, research institutions felt the pressure to ensure that their institutes maximised the outputs used to distribute structural funding. Funders also expressed feeling pressures. The growing number of applications for funding increased workload, generated internal stress, and built a constant struggle to find adequate peer-reviewers. Editors and publishers expressed a similar concern, stating that the pressure to publish and the current focus on quantity either pressured them to maximise their impact factor, or led them to receive more manuscripts than they could review and required them to use greater scrutiny to ensure the integrity of their publications. These additional efforts, in turn, forced them to increase article processing charges and subscription prices, both of which generated criticism from other actors. Finally, even policy makers and influencers were affected by the current pressures, in particular due to criticism regarding the distribution key in use to distribute funding between universities in Flanders. Because of its dependence on impact and output metrics,^2^ the distribution key was thought to be *“the reason that publications are so paramount in the assessments”* (PMI) within Flemish institutions. Despite the criticism towards performance-based research funding of research institutions, policy makers and influencers maintained that it was its inadequate use (i.e., using its parameters at the individual level) that was at the source of the problem. Consequently, even though the pressures and the culture of publish-or-perish were raised by nearly all interviewees, the root of the problem appears to be transferred from one actor to the next. This circle of blame further seemed to devalue individual responsibilities and actionable solutions, leaving most actors feeling frustrated and helpless. We will discuss this problem further in the section ‘A general resistance to change’.

##### Culture of profit and intolerance for failures and mistakes

In tight connection with the pressures and the culture of publish or perish, the emphasis towards a return on investment and outcomes was seen as potentially undermining the care and consideration that should be given to researchers themselves. This forgotten need for care easily links back to the lack of support faced by young researchers, the precariousness of research careers, and the lack of support for meeting integrity requirements, while it also explains the dominant intolerance for failure and mistakes. Interviewees from all actor groups spontaneously explained that failure, negative findings, and mistakes are almost invisible in science, despite being *very “important*”, “*valuable*”, and “*interesting*”. Intolerance for failure was even described as an incentive for falsification and detrimental practices. Despite praising the importance of disclosing mistakes, researchers who had had to deal with mistakes highlighted the discomfort and fear they engendered, especially in team settings where colleagues disagreed on the course of action they should take. The under-appreciation for negative results was also mentioned very frequently, with interviewees worrying that unpublished negative results wasted research resources. Still, researchers, research students, and lab technicians described negative results as highly frustrating, or as ‘*unlucky*’ (see the discussion on luck in [34]) and, besides rare exceptions, admitted that projects with negative results tended to be abandoned early, that experiments were often repeated until positive results were obtained, or that interpretations were curated (i.e., spin) to emphasise positive results (see relevant quotes in Supplementary file section 6). When asked about responsibility, interviewees once again seemed to pass the ball to one another. Researchers claimed that they were forced to look for positive findings since journals would not accept negative results and funders expected positive findings. Nonetheless, both journals and funders refuted this perspective, supporting that their true concern was the value and the quality of the work. Editors and publishers added that they rarely, if ever, received manuscripts with only negative results. One interviewee even told the story of a new journal dedicated to negative results which had to be shut down because it received “*no submission whatsoever*” (EP).

##### Unrealistic expectations

Intolerance for failure might be a simplistic expression of a bigger problem: science builds unrealistic expectations. Interviewees mentioned that too much was expected from researchers, potentially leading to frustrations, integrity deviations, or burn out. Different forms of expectations were perceived as being excessive and unrealistic. First, expectations of high yielding results, and extraordinary findings were considered to be embedded in the core of how science is evaluated. As we mentioned above, expectations of extraordinary findings imposed the wrong incentives on research practices. Such unrealistic expectations encouraged researchers to blend science and story-telling to embellish their findings. Second, expectations that researchers should work out of passion without personal benefits also surged from our interviews. Researchers were expected to work out of devotion without seeking personal gains or compensation. Many interviewees expected researchers to work outside ordinary schedules,^3^ to travel abroad regularly, and eventually even rethink their work–life balance to adapt to the demanding research life (see relevant quotes in Supplementary file section 6). Unfortunately, such expectations of personal sacrifice are not benign on researchers and research students. As we have briefly discussed above, several researchers who changed career explained that it was difficult to conjugate their professional and personal life, and that they felt the need to sacrifice the latter to ensure their professional survival. For many, the difficulty to keep a sane work–life balance played a significant role in their decision to move away from academia. But even those who suffered the effects of excessive expectations tended to perceive “*real scientists*” as those who should be passionate, devoted, and who give more than they could. Worrying about this unrealistic perspective and about the implications that unattractive research careers may cause in the long run, one policy maker or influencer advised that research careers and expectations should be revisited so that researchers do not have to *“sacrifice their wellbeing and their participation in the pleasures of a good economy just because they love science”.*

##### Competition, hierarchy, and unfair advantages

A few additional problems were linked to the social relationships which characterize the academic culture, such as competition, hierarchy, and advantages due to networking. The issue of competition raised mixed reactions from participants. On the one hand, some interviewees mentioned that competition was a necessary element of academia since it drove productivity and excellence while imposing limits on the authority of single researchers. Yet, most worried that competition was so strong that it challenged collaboration, collegiality, and openness. Not too far from competition, hierarchy was another problem mentioned by several interviewees. In discussing with research students and laboratory technicians, we understood that hierarchies were inherent to academia and that they deeply influenced interactions, openness, and integrity. For example, both technicians and PhD students mentioned that they would find it very difficult to openly criticize the conclusion or dubious behaviour of the principal investigator of a laboratory. Technicians mentioned that they would not dare to flag mistakes and errors because they felt that principal investigators had greater expertise and were “*much smarter*” than them. PhD students, on the other hand, said they wouldn’t dare to disagree with a supervisor’s inadequate practices (the example of gift authorship was used) because they were worried that the supervisor would “*make it hard*” on them and might not allow them to graduate. Researchers, on the other hand, criticized issues linked with inequities in statute and reputation between researchers, mostly related to career stage as we described above in the struggles of early career. Another relational component raised as a problematic was the unfair advantages that researchers can obtain from their networks. Networking is an inherent part of science and was mentioned many times as an essential factor for success [34]. To some, there was comfort in knowing that good relations can bring “*favours*” in case of need. Yet, most interviewees expressed discomfort and frustration about the advantages that networks and relations could yield, arguing that they often biased funding and publishing review processes. Refuting this idea, funders maintained that they make efforts to recruit international peers to minimize conflicting interests, while editors explained that the review process and the dependence on external reviewers’ assessments cancelled such biases. To substantiate this claim, editors provided examples where editors themselves have had their submissions rejected in their own journals. The discrepancy between the perspectives of researchers and the perspective of funders and editors makes this issue of unfair advantages difficult to resolve.

##### Punitive, not preventive

Adding to the above problems, a few interviewees worried that the scientific culture focused on punishment rather than on prevention of misconduct. Even though only a few interviewees mentioned this problem, their perspectives raised questions which are scarcely addressed in the current integrity discourse. A policy maker or influencer worried about the lack of a second chance for researchers convicted of misconduct. He explained that once misconduct is proven, universities generally ban or shame the convicted researcher without offering any later contact, support, or chance for retaliation. This interviewee believed that instead, institutions would benefit from rehabilitating deviant researchers and involving them to teach others how to avoid such mistakes. He believed that this would lead to higher relevance of integrity training, and would avoid that researchers who committed misconduct simply move on to a new university without any kind of follow up or notice — an issue that often happens in Europe where misconduct cases are not always disclosed publicly.

### A general resistance to change

In the final portion of our questions on research integrity, we asked researchers ‘who they believe was responsible for promoting integrity’. Although selected actor-specific responsibilities were mentioned, we quickly realised that integrity was generally seen as a shared responsibility in which all actors have a role to play.

“So I think that it’s a broad ecosystem, and everyone has a role to play in that.” (EP)

“I’m not going to say one person. I think it’s an extremely complicated theme, and extremely complicated idea, concept … So you cannot focus on one person. You need to target a lot of people.” (RCC)

“Everybody [laugh]. Everybody has their share of responsibility, of course.” (PMI)

This sharing of responsibilities, however, appeared to downplay individual responsibilities and to trigger a shared feeling of helplessness. For example, researchers believed that, to survive in the current system, they had to play by the rules of the game even if they disagreed with such rules. Institutions felt powerless on their own, and some interviewees even believed that it was unrealistic to believe in any drastic improvements.

“Everyone is behaving like this. Everyone is saying ‘Let's go for the safe road because this is how it is otherwise I will never get funding’, so …” (Researcher)

“One institution cannot change that.” (RIL)

“I don’t think we can expect, realistically speaking — but it’s cynical maybe — we can expect the great world change. It couldn’t change. You can try to make the ships sail a bit more in another direction but you cannot turn it. Therefore it’s too deep. The idea is … The views on what science is and how people work is too deep. It might be cynical if I’m saying it now.” (RCC)

Lacking the empowerment or hope to take action, interviewees tended to transfer the root of the problems from one to another, creating a circle of blame which fostered frustration and distrust between actor groups. For instance researchers had to cut corners because research institutions pressure them to publish; research institutions had to push researchers to publish because policy makers distribute structural funding based on publication and research outputs; policy makers had to distribute funding based on publications because society wants a return on its investment, etc. In other words, each actor appears to use the failures of higher actor groups to justify its personal inability to endorse best practices. But the complex interplay between actors also led to smaller circular criticism. For example, researchers criticized funders for evaluating them on quantity rather than on quality. But funders explained that even when they have policies in place to ignore quantity, peer-reviewers — who are themselves researchers — tended to cling to old quantitative metrics. Similarly, universities criticized that journals looked for hype rather than quality, but journals believed that the real problem was that universities used journals to evaluate researchers, not the decisions that journals take on what they choose to publish. Given the popular perspective that science is built around a community where all actors share the common goal of advancing knowledge, internal distrust and lost hopes for true change are necessarily a worry for the future.

## Discussion

The present paper reveals a rich account of various stakeholders’ perspectives on the problems that affect research integrity and research culture. The paper complements associated findings on definitions and attribution of success in science [34]. While it is technically impossible to integrate all diverse and sometimes inconsistent responses in a well-structured discussion, we would like to highlight three main findings from our paper series which provide insights for the next steps towards better science. Specifically, we wish to show how our findings align with the pressing need to i) revisit research assessment, ii) tackle inadequate climates, and iii) foster inter-actor dialogue.

First, it is clear that the problems and frustrations raised by our respondents were intimately connected to the way success is attributed in science [34]. Pressures, incentives, expectations, and competition are all related to the way in which researchers are assessed. Consequently, the revision of research assessment needs to become central to the integrity discourse. In the associated paper, we show that part of the criticism against current assessments resides in their overreliance on reductionistic metrics [34]. While we understand the strong emphasis on metrics from a pragmatic point of view, in practice, our participants considered reductionistic metrics as imprecise, disruptive, and at the very heart of most problems afflicting science. Without discrediting excellent science that yields remarkable metrics, we must recognise that excellent science does not necessarily translate into such metrics and that current output metrics provide, at best, a reductionist picture of the qualities and merits of researchers. The lack of consideration for important research processes such as openness, transparency, and collaboration may dissuade researchers from investing in such practices which are ultimately essential for the quality and the integrity of science. In current research climates, researchers who commit to good science regardless of short term impactful outputs might even place their career at risk. Wide-spread expectations of extraordinary results further add to the problem, not only by suggesting that extraordinary science should be the norm — a paradox in itself — but also by devaluing negative findings and small-steps-science, both of which are key to advancing knowledge. And yet, current assessments were also said to ignore — even inhibit — high risk innovation, originality, and diversity. Considering all this, it is obvious that research assessments must be addressed. A number of recent initiatives, such as the Declaration on Research Assessment (DORA [46];), the Leiden Manifesto [47], the Metric Tide [48], the Hong Kong Principles for Assessing Researchers [49], and numerous scientific editorials and public fora (e.g., [50–53]) are important pioneers in exposing the challenges of current assessments. Our findings echo these challenges and further link current problems to research integrity, thereby reinstating that research assessment must become central to the discourse on research integrity.

Second, our findings suggest that approaches to foster integrity should focus on changing research climates rather than on individual behaviours. Our findings are far from the first to highlight the crucial role that research climates play on research practices and integrity ([23–32], or more details in [33]). Still, in today’s academia, the majority of approaches aiming to tackle integrity issues capitalise on personal behaviours rather than on systemic problems behind faulty research climates [33]. This person-centred perspective has profound implications on the way we perceive integrity. On the one hand, it implies that research integrity is predominantly a responsibility of researchers rather than a shared responsibility of different stakeholders. In fact, codes of conduct, integrity courses, whistleblowing channels, and internal oversight all capitalize on individual researchers. Without discrediting the value of these approaches in building awareness around research integrity, our findings support that other actors also have a crucial role to play in reshaping research climates. Important initiatives from institutions, funders, and policy advising groups are stepping stones in extending the responsibilities for research integrity (e.g., [54–56]). The recommendation from such reports, however, need to lead to concrete changes in practices and policies. On the other hand, the person-centred perspective of integrity also ignores the pervasive dissonance between what researchers know they should do (i.e., integrity) and what they need to do to survive in their career (i.e., success). Our findings suggest that research careers are precarious, highly competitive, and that they can impact the wellbeing of researchers. Young researchers in particular often lack the resources, guidance, and support they need to flourish or simply to migrate smoothly to careers outside academia (also see [57–59]). Adequate support for researchers, including through an attentive consideration of their well-being and struggles, should be an immediate priority within research institutions. In the long term however, funders, institutions, and policies will need to work together to address the imbalance between junior and senior career opportunities as well as the problematic high-expectation, short-term, and insecure contracts that dominate early research careers. The tenacious culture that currently disrupt integrity will only change if researchers are given a chance to survive the system without feeling the need to compromise integrity.

Finally, our findings reinforce the need for inter-actor dialogue in discussions on research integrity. When describing success in science, we argue that a comprehensive inter-actor dialogue is needed to combine different meanings and expectations of scientific success [34]. Similarly, when discussing problems of science with multiple actors, we understood not only that perspectives differ from one actor to the next, but that the lack of inter-actor discussion leads to a circle of blame in which no one feels able to tackle the problems. Even though actors depend on one another, the opportunities to discuss and share decisions between them are limited, especially for early career researchers. This segregation leads to misunderstandings, false beliefs, and missed opportunity for joint actions. As researchers, we were ourselves surprised to realise that pressures also affect institutions, funders, editors, and policy makers. We thus believe that the best way forward is to create fora for participatory decisions on topics of success, assessments, climates, and integrity. Prioritising opportunities for inter-actor dialogue and actively seeking the voice of overlooked actors will help reduce victimisation and blame and promote well-considered joint action.

### Study limitations

A few points are important to consider when interpreting our findings. First, when discussing a topic such as research integrity, participants may feel that they have to defend or conceal the practices in place at the organisation where they work, resulting in a possible lack of transparency. Ensuring confidentiality is essential to obtain transparent answers. To minimize risks of identification, we grouped responses by general actor group rather than by individual participants, and decided that any potentially damaging information revealed during our interviews or focus groups would remain confidential, even if it revealed possible misconduct. After the focus group discussions, researchers, research students, and laboratory technicians were given a list of contacts where they could safely declare or discuss possible misbehaviours, but the research team preserved full confidentiality on possible misconduct revealed within this project and did not intervene further. Consequently, although we cannot guarantee the accuracy and transparency of participants’ response, we ensured that participants felt confident that they could be honest without risk.

A few general limitation also applied to both portions of this study (i.e., also included in [34]). First, given the exploratory and qualitative nature of this project, our sample is neither exhaustive nor fully representative. We chose to ask for personal perspectives rather than official institution or organisation views since we believed it would allow us to capture genuine beliefs and opinions and avoid rote answers. We thus encouraged participants to share their personal thoughts rather than the thoughts that could be attributed to their entire actor groups, institution, or organisation. We considered that these personal beliefs and opinions are crucial in shaping the more general views of organisations, yet we urge our readers to remain careful when making group comparison and generalisations.

Adding to the above concern, it is important to keep in mind that the research world is complex and not organized in distinct actor groups. Participants could often fit in more than one category by endorsing several research roles. We asked all participants whether they agreed with the category we assigned them in, and we refined and exemplified the definitions of our actor groups to reflect the participants’ distinctions. Yet, we had to consider each actor category not as a closed box with characteristic opinions, but as a continuum which may or may not hold divergent views from other actor groups. Our findings helped capture views which may have been overlooked in past research which focused on researchers, but should not be used to discriminate or represent the opinions of entire actor groups.

Finally, it is important to consider that given the richness of the information gathered, certain findings may be displayed with greater importance than others simply based on the authors’ personal interests. We were careful to include also the views we disagreed with or found to be of limited interest, yet it is inevitable that some of the selection and interpretation of our findings was influenced by our own perspectives. To maximise transparency on the genuine views of our informers, we supplement our interpretation of the findings with quotes whenever possible, most of which are available in the supplementary file.

## Conclusions

Our findings shed light on the complex interplay between success, research culture, and research integrity. Involving not only researchers, but a wide range of actors who hold different roles in science, we showed that there is great tension between what researchers should do to advance science, and what they had to do to be successful. This finding resonates with debates that have been taking place in the past few years. But despite heated discussion, initiating changes in research climates takes time, effort, and broad coordination. Our findings extrapolate a few action points which might help coordinate such changes. First, assessments of success must be tackled and must become central to the integrity debate. Second, approaches to promote better science should be revisited to address the impact that research climates have on research practices and research integrity rather than to capitalize on individual researcher’s compliance and personal responsibility. Finally, inter-actor dialogues and shared decision making are crucial to building joint objectives for change. Such dialogues should actively seek the voices of parties which are forgotten from the current discourse, and should genuinely aim to construct a collective understanding of the problem so that actors can join forces for change.

## Supplementary Information

**Supplementary file 1.**

## Data Availability

**Data**: Given the sensibility and risk of identification of qualitative data, we are unable to share full transcripts. In the manuscript however, we attempted to be as transparent as possible by providing numerous quotes in the text and in tables to exemplify and support our claims. **Materials**: Our focus group and interview guides, as well as the consent forms and information sheet we shared with participants are available on the registration of this project [35].

## Abbreviations

BOF: Bijzonder Onderzoeksfonds [special research funds]
EP: Editor(s) or publisher(s)
FA: Funding agency(s)
LT: Laboratory technician(s)
PMI: Policy maker(s) or influencer(s)
PostDoc: Post-Doctoral researcher(s)
QRP: Questionable research practices
RCC: Researcher(s) who changed career
RIL: Research institution leader(s)
RIN: Research integrity network member(s)
RIO: Research integrity office member(s)

## Footnotes

1 In Flanders, an important portion of the federal funding for research institutions is distributed using using performance-based calculations [41]. The BOF (bijzondere onderzoeksfonds [special research funds]) key was most mentioned by our interviewees, but a portion of the core structural funding of universities is also distributed based on each universities’ performance. Both distribution calculations are highly dependent on output indicators [42]. In the distribution for the BOF funding, publication metrics account for over 40% of the final score for distribution [43]. Similar funding distribution systems exist in other countries around the world [44, 45].

2 Since the interviews have been conducted, a new version of the BOF-key has been developed and released (mid 2019). Nonetheless, a large proportion of the resource allocation distributed through the new BOF-Key still depends on output metrics and publications with an impact factor.

3 Working beyond the 9–5 schedule was even seen as a factor for success [34].

## References

[1] Pupovac V, Fanelli D (2014). Scientists admitting to plagiarism: a meta-analysis of surveys. Sci Eng Ethics.

[2] Martinson BC, Anderson MS, De Vries R (2005). Scientists behaving badly. Nature..

[3] Fanelli D (2009). How Many Scientists Fabricate and Falsify Research? A Systematic Review and Meta-Analysis of Survey Data. PLoS One.

[4] National Institute of Health. Research Misconduct - Definitions 2018 [Available from: https://grants.nih.gov/policy/research_integrity/definitions.htm.

[5] Bouter LM, Tijdink J, Axelsen N, Martinson BC, ter Riet G (2016). Ranking major and minor research misbehaviors: results from a survey among participants of four world conferences on research integrity. Res Integrity Peer Rev.

[6] Antes AL, Brown RP, Murphy ST, Waples EP, Mumford MD, Connelly S (2007). Personality and ethical decision-making in research: the role of perceptions of self and others. J Empir Res Hum Res Ethics.

[7] Bailey CD (2015). Psychopathy, academic accountants' attitudes toward unethical research practices, and publication success. Account Rev.

[8] Brown RP, Tamborski M, Wang X, Barnes CD, Mumford MD, Connelly S (2011). Moral credentialing and the rationalization of misconduct. Ethics Behav.

[9] Davis MS, Riske-Morris M, Diaz SR (2007). Causal factors implicated in research misconduct: Evidence from ORI case files. Sci Eng Ethics.

[10] Davis MS, Wester KL, King B (2008). Narcissism, entitlement, and questionable research practices in counseling: a pilot study. J Couns Dev.

[11] Hren D, Vujaklija A, Ivanišević R, Knežević J, Marušić M, Marušić A (2006). Students' moral reasoning, Machiavellianism and socially desirable responding: implications for teaching ethics and research integrity. Med Educ.

[12] Miller A, Shoptaugh C, Wooldridge J (2011). Reasons not to cheat, academic-integrity responsibility, and frequency of cheating. J Exp Educ.

[13] Okonta P, Rossouw T (2013). Prevalence of scientific misconduct among a group of researchers in Nigeria. Dev World Bioethics.

[14] Fang FC, Bennett JW, Casadevall A. Males are overrepresented among life science researchers committing scientific misconduct. MBio. 2013;4(1):e00640–12. 10.1128/mBio.00640-12. 10.1128/mBio.00640-12 PMC3551552 23341553

[15] Ghias K, Lakho GR, Asim H, Azam IS, Saeed SA (2014). Self-reported attitudes and behaviours of medical students in Pakistan regarding academic misconduct: a cross-sectional study. BMC Med Ethics.

[16] Fanelli D, Costas R, Larivière V (2015). Misconduct policies, academic culture and career stage, not gender or pressures to publish, Affect Scientific Integrity. PLoS One.

[17] Kraemer Diaz AE, Spears Johnson CR, Arcury TA (2015). Perceptions that influence the maintenance of scientific integrity in community-based participatory research. Health Educ Behav.

[18] Babalola YT (2012). Awareness and incidence of plagiarism among undergraduates in a Nigerian Private University. Afr J Libr Arch Inf Sci.

[19] Adeleye OA, Adebamowo CA (2012). Factors associated with research wrongdoing in Nigeria. J Empir Res Hum Res Ethics.

[20] Wright DE, Titus SL, Cornelison JB (2008). Mentoring and research misconduct: an analysis of research mentoring in closed ORI cases. Sci Eng Ethics.

[21] Buljan I, Barać L, Marušić A (2018). How researchers perceive research misconduct in biomedicine and how they would prevent it: a qualitative study in a small scientific community. Account Res.

[22] Mumford MD, Devenport LD, Brown RP, Connelly S, Murphy ST, Hill JH (2006). Validation of ethical decision making measures: evidence for a new set of measures. Ethics Behav.

[23] Davies SR (2019). An ethics of the system: talking to scientists about research integrity. Sci Eng Ethics.

[24] Wester K, Willse J, Davis M (2010). Psychological climate, stress, and research integrity among research counselor educators: a preliminary study. Couns Educ Superv.

[25] Tijdink JK, Verbeke R, Smulders YM (2014). Publication pressure and scientific misconduct in medical scientists. J Empir Res Hum Res Ethics.

[26] Singh HP, Guram N (2014). Knowledge and attitude of dental professionals of North India toward plagiarism. North Am J Med Sci.

[27] Fanelli D (2010). Do pressures to publish increase scientists' bias? An empirical support from US states data. PLoS One.

[28] Anderson MS, Ronning EA, De Vries R, Martinson BC (2007). The perverse effects of competition on scientists' work and relationships. Sci Eng Ethics.

[29] Shrader-Frechette K (2011). Climate change, nuclear economics, and conflicts of interest. Sci Eng Ethics.

[30] Lundh A, Krogsbøll LT, Gøtzsche PC (2012). Sponsors’ participation in conduct and reporting of industry trials: a descriptive study. Trials..

[31] Kaiser KA, Cofield SS, Fontaine KR, Glasser SP, Thabane L, Chu R (2012). Is funding source related to study reporting quality in obesity or nutrition randomized control trials in top-tier medical journals. Int J Obes.

[32] DuBois JM, Anderson EE, Chibnall J, Carroll K, Gibb T, Ogbuka C (2013). Understanding research misconduct: a comparative analysis of 120 cases of professional wrongdoing. Account Res.

[33] Aubert Bonn N, Pinxten W (2019). A decade of empirical research on research integrity: what have we (not) looked at?. J Empir Res Hum Res Ethics.

[34] Aubert Bonn N, Pinxten W. Rethinking success, integrity, and culture in research (part 1) - a multi-actor qualitative study on success in science. Res Integrity Peer Rev. in revie. 10.1186/s41073-020-00104-0 PMC7807516 33441187

[35] Aubert Bonn N, Pinxten W. Rethinking success, integrity, and culture in science (Re-SInC). Open Science Framework, 2016. 10.17605/osf.io/ap4kn.

[36] Nuffield Council of Bioethics (2014). The culture of scientific research in the UK.

[37] Learning S, Wellcome Trust (2020). What researchers think about the culture they work in.

[38] Metcalfe J, Wheat K, Munafò M, Parry J (2020). Research integrity: a landscape study.

[39] Elo S, Kyngas H (2008). The qualitative content analysis process. J Adv Nurs.

[40] Dierckx de Casterlé B, Gastmans C, Bryon E, Denier Y (2012). QUAGOL: a guide for qualitative data analysis. Int J Nurs Stud.

[41] Engels TCE, Guns R (2018). The Flemish performance-based research funding system: a unique variant of the Norwegian model. J Data Inf Sci.

[42] Peters A (2019). Public funding of universities, and indicators used in the funding systems : a Euregional perspective.

[43] Decreet betreffende de organisatie en financiering van het wetenschaps en innovatiebeleid [Decree concerning the organization and financing of science and innovation policy], Art. 63/1 (2009).

[44] Franzoni C, Scellato G, Stephan P (2011). Changing incentives to publish. Science..

[45] Zacharewicz KJT. Research Performance Based Funding Systems: a Comparative Assessment. European Commission; 2016. Report No.: ISBN 978–92–79-68715-0 - ISSN 1831–9424. Available from: http://publications.jrc.ec.europa.eu/repository/bitstream/JRC101043/kj1a27837enn.pdf.

[46] American Society for Cell Biology (2013). San Francisco Declaration on Research Assessment.

[47] Hicks D, Wouters P, Waltman L (2015). Rijcke Sd, Rafols I. the Leiden manifesto for research metrics. Nature News.

[48] Wilsdon J, Allen L, Belfiore E, Campbell P, Curry S, Hill S (2015). The Metric Tide: Report of the Independent Review of the Role of Metrics in Research Assessment and Management.

[49] Moher D, Bouter L, Kleinert S, Glasziou P, Sham MH, Barbour V, Dirnagl U. The Hong Kong Principles for assessing researchers: Fostering research integrity. PLoS Biol. 18(7):e3000737. 10.1371/journal.pbio.3000737. 10.1371/journal.pbio.3000737 PMC7365391 32673304

[50] A kinder research culture is possible. Nature. 2019;574:5–6. 10.1038/d41586-019-02951-4 31576053

[51] Holtrop T (2018). LSE Impact Blog.

[52] Gadd E (2018). LSE Impact Blog.

[53] Bryce C, Dowling M, Lucey B (2018). Times Higher Education.

[54] Martinson BC, Thrush CR, Lauren CA (2013). Development and validation of the survey of organizational research climate (SORC). Sci Eng Ethics.

[55] The Wellcome Trust and Shift Learning (2020). What Researchers Think About the Culture They Work.

[56] Saenen B, Borell-Damián L. EUA briefing - reflections on university research assessment: key concepts, issues and actors: European Universities Association; 2019. Available from: https://eua.eu/resources/publications/825:reflections-on-university-research-assessment-key-concepts,-issues-and-actors.html.

[57] Woolston C (2017). Graduate survey: a love–hurt relationship. Nature..

[58] Van de Velde J, Levecque K, Mortier A, De Beuckelaer A. Waarom doctorandi in Vlaanderen denken aan stoppen met doctoreren [Why PhD students in Flanders think about stopping their PhDs]. ECOOM Brief (no 20). 2019; Available from: http://hdl.handle.net/1854/LU-8630419.

[59] Heffernan TA, Heffernan A (2019). The academic exodus: the role of institutional support in academics leaving universities and the academy. Prof Dev Educ.

