# Supplementary material for: Rethinking success, integrity, and culture in research (part 2) — a multi-actor qualitative study on problems of science
Source: Res Integr Peer Rev. 2021 Jan 14;6:3. doi: 10.1186/s41073-020-00105-z (PMC7807493; doi:10.1186/s41073-020-00105-z)
Supplement: Supplementary file 1 [file 41073_2020_105_MOESM1_ESM.pdf]

Supplementary file

Rethinking success, integrity, and culture in research (part 2) — A multi-actor qualitative study on problems of science

Noémie Aubert Bonn, Wim Pinxten

Table of contents

1. RESEARCH TEAM AND REFLEXIVITY 2

2. COREQ CHECKLIST 4

3. GENERAL INTERVIEW GUIDE 6

4. GENERAL FOCUS GROUP GUIDE 8

5. REASON FOR TAKING INTEGRITY ISSUES SERIOUSLY 11

6. QUOTES FOR THE PROBLEMS OF SCIENCE 13

1. RESEARCH TEAM AND REFLEXIVITY

In accordance with the COnsolidated criteria for REporting Qualitative research checklist (COREQ; page 4), and in respect of transparency, we found important to provide further characteristics about the setting and the interviewer at the time of the study.

Besides one early interview with an institution leader in which WP, assistant professor, attended to provide feedback about the interview, all other interviews and focus groups were conducted by NAB, with no additional non-participant or assistant.

NAB is a female PhD student in the Faculty of medicine and life science of Hasselt University, Belgium, with a background in cognitive neuroscience and bioethics. Coming from Canada, NAB had the advantage of bringing a certain neutrality in the interviews by not being strongly affiliated with one or another Flemish region, and by not corresponding to an established research group.

Before conducting the interviews and focus groups, NAB followed courses about developing interview questions, conducting focus groups, and analysing qualitative data offered from Flemish universities and from the Flanders' Training Network for Methodology and Statistics (FLAMES). In addition, she used the resource books from the Focus Group Kit by Richard A. Krueger and David L. Morgan (1), and discussed with RDV — expert in qualitative inquiries and part of the team that built the original guide upon which we inspired ours — to gain insight on building, conducting, and analysing focus groups and interviews.

Besides a few exceptions, NAB had no prior relationship with most participants, and the first contacts were established with the invitation email. No repeat interviews were carried out. Before the interview, NAB described the project briefly and explained the purpose of the interview informally. On some occasions where interviewees were anxious to know more about the project in advance, NAB would email the main themes targeted, but would not share the interview guide with participants by fear that this may lead to rote answers.

Bias and assumptions

NAB holds the view that research integrity is largely determined by the research system, and the interview guide was necessarily not unbiased to this perspective. Nonetheless, if participants shared a different view (e.g., if they believed that integrity was solely a matter of personality), NAB was careful not to contradict or bias interviewees' ideas towards her perspective. In re-reading quotes with the research team, we were careful for possible misinterpretations, and when quotes were interpreted differently by WP or RDV, we adapted the nodes and interpretations to make sure they fit the words of the participants. Both WP and RDV helped in classifying the main nodes into general categories of *Who*, *What*, *How*, and *Luck*. Initially, we were tempted to classify these four categories in *Products* of success (the What) and *Potential* for achieving success (the Who, How, and luck). However, after several discussions, we realized that doing so may reinforce the perspective that products are the ones which truly indicate success, while potentials are simply increasing the chance of yielding better products. As we describe in our extended findings, many of our interviewees considered the *Who* and especially the *How* to be genuine successes in themselves. In this regard, we intentionally kept the four categories together as each representing successes in themselves.

Study design and interview/focus group setting

Interviews and focus groups were conducted in private meeting rooms or offices or, according to preference, in public spaces (N=2) or through video call (N=3). One of the interview conducted through video call had some sound and connection problems, but the other video calls went very smoothly.

Interviews lasted on average 60 minutes, depending on the time granted by the interviewee (range from 34 to 80 minutes). Focus groups lasted around 120 minutes each and included a five-minute break.

All interviews were audio recorded and transcribed verbatim by the interviewer (NAB) or a university-approved transcription service. Transcripts were not returned to participants except in select cases where participants expressed a wish to monitor their answers, and in cases where the quotes of interest might have jeopardized the confidentiality of participants. No repeat interviews were undertaken. After most interviews, the interviewer filled a self-questionnaire about the interview to note any abnormalities and general feelings of the interview data.

Reference

1. Morgan, D. L., & Krueger, R. A. (1998). London: Sage Publications.

2. COREQ CHECKLIST

COnsolidated criteria for REporting Qualitative research

Topic	Item No.	Guide Questions/Description	Reported in section:
**Domain 1: Research team and reflexivity**			
Personal characteristics			
Interviewer/facilitator	1	Which author/s conducted the interview or focus group?	Supp. file
Credentials	2	What were the researcher's credentials? E.g. PhD, MD	Supp. file
Occupation	3	What was their occupation at the time of the study?	Supp. file
Gender	4	Was the researcher male or female?	Supp. file
Experience and training	5	What experience or training did the researcher have?	Supp. file
Relationship with participants			
Relationship established	6	Was a relationship established prior to study commencement?	Supp. file
Participant knowledge of the interviewer	7	What did the participants know about the researcher? e.g. personal goals, reasons for doing the research	Supp. file
Interviewer characteristics	8	What characteristics were reported about the inter viewer/facilitator? e.g. Bias, assumptions, reasons and interests in the research topic	Supp. file
**Domain 2: Study design**			
Theoretical framework			
Methodological orientation and theory	9	What methodological orientation was stated to underpin the study? e.g. grounded theory, discourse analysis, ethnography, phenomenology, content analysis	'Methods' → 'Analysis'
Participant selection			
Sampling	10	How were participants selected? e.g. purposive, convenience, consecutive, snowball	'Methods' → 'Participants'
Method of approach	11	How were participants approached? e.g. face-to-face, telephone, mail, email	Supp. file
Sample size	12	How many participants were in the study?	Table 1
Non-participation	13	How many people refused to participate or dropped out? Reasons?	—
Setting			
Setting of data collection	14	Where was the data collected? e.g. home, clinic, workplace	Supp. file
Presence of non-participants	15	Was anyone else present besides the participants and researchers?	Supp. file
Description of sample	16	What are the important characteristics of the sample? e.g. demographic data, date	Table 1
Data collection			
Interview guide	17	Were questions, prompts, guides provided by the authors? Was it pilot tested?	Supp. file
Repeat interviews	18	Were repeat inter views carried out? If yes, how many?	Supp. file

Audio/visual recording	19	Did the research use audio or visual recording to collect the data?	Supp. file
Field notes	20	Were field notes made during and/or after the interview or focus group?	Supp. file
Duration	21	What was the duration of the inter views or focus group?	Supp. file
Data saturation	22	Was data saturation discussed?	—
Transcripts returned	23	Were transcripts returned to participants for comment and/or correction?	Supp. file
**Domain 3: analysis and findings**			
Data analysis			
Number of data coders	24	How many data coders coded the data?	Page 7-8
Description of the coding tree	25	Did authors provide a description of the coding tree?	Figure 1 and Figure 3 in (1)
Derivation of themes	26	Were themes identified in advance or derived from the data?	'Methods' → 'Analysis' and Supp. file
Software	27	What software, if applicable, was used to manage the data?	'Methods' → 'Analysis'
Participant checking	28	Did participants provide feedback on the findings?	Supp. file
Reporting			
Quotations presented	29	Were participant quotations presented to illustrate the themes/findings? Was each quotation identified? e.g. participant number	Throughout
Data and findings consistent	30	Was there consistency between the data presented and the findings?	Throughout
Clarity of major themes	31	Were major themes clearly presented in the findings?	Figure 1 and Figure 3 in (1)
Clarity of minor themes	32	Is there a description of diverse cases or discussion of minor themes?	Throughout

Reference

1. Aubert Bonn N, Pinxten W. Rethinking success, integrity, and culture in research (part 1) - A multi-actor qualitative study on success in science. Research Integrity and Peer Review. 2020.

3. GENERAL INTERVIEW GUIDE

A part of my research is to explore the views of different actors that contribute to the research system.

To protect your privacy, I want to avoid disclosing your specific job title and to place you in one of bigger category of research actors. I may use a higher level of details to describe the type of participants included in each category, but I won't link direct quotes with company or institution names.

I placed you in the category ***actor group***. Does that sound good to you?

Introduction and information on respondent's career

1. Before anything, I would like you to **describe your work** to me, in your own words.

   Prompt: In a broader perspective, what would you say is your role is in the scientific system?

2. In this job, you obviously **care for scientific excellence**. How would you say you fulfil this goal in your work?

   We will get back to this a bit later. For now, I will change topic and I want us to talk about success as this is an important topic that we are trying to understand in the project.

Success in science

3. First, try to think about scientists you've known that were very successful. What do you think made these scientists **successful**?

   Prompt: Which characteristics do you think are most important to advance a researcher's career?

4. Do you feel like these characteristics are **captured in current research assessments** and evaluations? In which ways?
5. (If time allows) What do you feel that your actor group **should do to promote successful science**? Do you see that happening?

Tensions or conflict between success and integrity

6. You mentioned that X, Y, Z are criterions that indicate success in research. Do you think that these are **also indicators of quality**? Sound research?

   *Prompt: Which criterions do you think **indicate the quality** of the research?*
   *Prompt: Which criterions do you feel are **not suited** to indicate the quality of the research? Explain.*

7. Does it happen that you see excellent researchers but for some reason these researchers **don't succeed in getting ahead** with their career? Can you give me some examples?
8. Do you feel that the way in which success is attributed allows to for **emerging scientists** to become successful?

Current problems

Let's change the topic now; leave aside success for a bit and look at when science is not at its best. Like I said, I am not here to denounce or condemn cases, so I will make sure to protect the confidentiality of cases you may discuss.

9. Have you ever had to **deal with science** which you considered was **not really in line with the rules of science**? What happened?

10. Can you give me precise examples of the elements that you consider **signs of bad or sloppy research**? What are **red flags**?

Motives for bad practices

11. **Why** do you think bad research practice happens?
12. Do you think **anyone could** end up in such a situation or only types of people?

Responsibilities towards integrity

We have already discussed how to promote successful science, now I would like to gather your thoughts on how to prevent sloppy research.

13. What do you think should be done to **prevent bad science** from happening?
14. **Who** should take the lead to make these changes happen? Who else should be involved?
15. What do you consider is the **responsibility of your *actor group*** to protect integrity?
16. Where does your responsibility **end**?

One change

Finally, if you could pick **one important change that needs priority** right now in how research works, what would it be? How do you think this change could be done?

(if time allows) Alternatives

17. If there were no rules for evaluating scientists, and you could **start from scratch**, what would you like to look at when assessing scientists?

    *Prompt: What are the characteristics that **YOU think are most important** for researchers to do good research?*

4. GENERAL FOCUS GROUP GUIDE

Intro:

1. Who you are
2. What is your area of research
3. Describe a typical day of work
4. What's your favourite ice cream flavor

Scientific career

Before starting, I would like to know a little more about your career as a researcher.

18. Specifically, I would like to know what is it that makes your work so great? What do you feel is most satisfying, most rewarding about your career?

Prompt: When people ask you why you chose to be a researcher, what first comes to mind?

We will get back to this a bit later. But for now I will change topic and I want us to talk about success.

Success in science

19. Think of a person in your field who you think is **very successful**. (It doesn't have to be one person in particular, it can just be some characteristics of many different people, can be yourself in 20 years…) How do researchers **become successful**? What, in your view makes this person a success?

Prompt: What are the most important factors for advancing in your career?
Prompt: What are the funders and the employers looking at?

20. Now imagine that I am a newcomer in your field and I ask you what I must do to stay on the top, **what would you tell me**?

So you say that successful scientists are generally scientists who do X, Y, Z.

21. Do these successful scientists **reflect or mirror the kind of scientist you want to be**? Do you have such aspirations for success?

Tensions or conflict between success and integrity

22. As we discussed, you point out that funders and employers look at X, Y, Z… Do you think that **these criterions for success indicate outstanding or excellent research** (e.g., appropriate methods, relevant topic, high quality work)?

*Prompt: Which criterions do you think **indicate the quality** of the research?*
*Prompt: Which criterions do you feel are **not suited to indicate the quality** of the research? Explain.*

23. Now try to think of a **colleague** who, in your opinion, **does good research but cannot reach success in science**?

Prompt: *What in your opinion explains that this researcher cannot reach a successful career?*

24. **What would you say to this researcher to help him/her get ahead**?

Current problems

Let's change back the topic now; leave success aside for a bit and discuss what it is like to be a researcher. So you remember that at the beginning of the discussion, I asked you about the aspects of research that make you like your career. Now I want us to talk about the other side of things, about what **frustrates** you as a researcher.

25. So let's say I am a **newcomer** in your field. I just started working in your lab and I am not sure whether I should follow a scientific career. If I asked you what are the **most frustrating things about working in science**, what would you tell me?

    Prompt: what would you tell me are some of the biggest frustration I could encounter?

26. All right, so as we have discussed, being a researcher is not necessarily always easy. It can sometimes happen that things go really wrong. Have you **ever seen or heard** of a situation in which you thought research was conducted in a way that was **against the 'rules' of science'**? What happened? What did/would you do?

Motives for bad practices

27. **Why** do you think researchers were acting in this way?
28. Do you think **any researcher could end up** in such a situation?

CURRENT VIEWS ON RESPONSIBILITY

29. **What** do you think should be done to **prevent bad science** from happening?
30. **Who should take the lead** to make these changes happen? Who else should be involved?
31. **What can you do**?

    Prompt: Do you feel like you miss something to be able to change things yourself?

Solutions

To finish, I would like to ask a more concrete question.

32. Finally, if you could pick **one important change that needs priority** right now in the research system, in how science works, what would it be?

    Prompt: How do you think this change could be done?

Personal success

Now before we finish, I want you to think back about the discussion we have had on success, and on criterions that are most often used to evaluate a research career. But now, I would like you to think about yourself as a researcher, and to think about your strength, about what makes you feel accomplished in your work. **What do you think is your biggest contribution to your work**, or **something that you think is key to be a good researcher**, regardless of the criterions we have said before. (For example, maybe you think that the fact that you brush your teeth after lunch is key to the success of your research team.)

I will not ask you to discuss it this time, but I would like everyone to take one of these little pieces of paper. On the piece of paper, I would like you to make up 3 to 5 criterions for funders and employers. I want you to think about what you consider your biggest contributions in your work, and to make up criterions you would think, if funders and employers evaluated, you would have better chances to succeed.

Summary

Is there anything you would like to mention that we failed to discuss today?

5. REASON FOR TAKING INTEGRITY ISSUES SERIOUSLY

Select quotes to show different reasons to take integrity issues seriously

Sample quote	actor
**CHANGING THE COURSE OF SCIENCE**	
I suspect that those tiny [fraudulent] publications, they will get falsified, but nobody puts too much attention to that anyway, **they are not moving the real direction of science, you know, this is such a small study somewhere, it doesn't really matter that much** if it turns out to be incorrect. [...] Cutting corners sometimes is not changing the course of science. [...] So it can happen to everybody that you cut corners. It's just, is that a scientific corner or is that an esthetic thing? But everybody will be tempted at some stage to do that so let's just hope that the majority of people will have the scientific integrity not to do that. But... (sighs) I think, certainly with PhD students I only hear transiently... But I don't think you can get a full article based on one little corner that has been cut. I think you really need to cross that line, and then go up and not do that again. I think that's where things go wrong when it becomes a standard, where you've made it once and realised it doesn't make a difference...	RIL
**CHANGING CONCLUSIONS**	
In my lab if I look, the only misconduct I've picked up was just stupidity. PhD students who scanned a little too short and had to go back to the scanner and thought 'I could just copy-paste the bottom bit because there's nothing on it anyway'. **That's real misconduct, but at the same time, that's not scientific fraud. Well it was, it is scientific fraud, but he was not changing a conclusion**, he was just too lazy to scan a really nice experiment [...] What I consider cheating is that you leave out the data that don't suit your model. Or you make up data to get your model correctly. That is what I call cheating.	RIL
It's difficult to prove intention, and **for us that's not that important. If it's actually a deception, doesn't matter if it's intentional or not**. Then we need to correct the literature if it's published. So you know, again I think a lot of the misconduct investigation that institutions do, they put a lot of emphasis on the intentional bit because that's part of the employer status and so on. Whereas we as journals are not that interested in that part. **We're really interested in 'Is this research trustworthy or not?**	EP
Intent is something that editors are not in a position to properly evaluate. [...] And this is where they have to rely on institutions to determine, to really establish and ascertain whether there is misconduct or not. Where the responsibility of the editor is in **correcting the scientific record**. And that, it doesn't matter whether it's misconduct or not in a way. [...] **if something is wrong**, and you're unsure as an editor **whether it's misconduct or not, it doesn't matter**. It also needs to be corrected in the scientific record.	EP
I think usually that's something that is important in evaluating those cases. It's really, does the act, the problem that you have identified, **does that actually lead to a different... To change the nature of the conclusion?** And really make the data say something different than what it says? And so… You know that tends to be misconduct.	EP
**POOR QUALITY OF FINDINGS**	
You always have to take into account the rules, the procedures of good research.[...] If you want to talk about society, your reference group must be big enough so it can be a reference, a real reference to the society. So if you don't put up the research in advance in a good way, then **it's also sloppy or bad research. Because all the results that come out of this, even if they're positive, they won't be representative for the reality.** And that can lead to – if it's research in medicine–that can be very dramatic. So that's sloppy research.	PMI
**MISUSE OF RESEARCH MONEY**	
[During a discussion about copying deliverables for a different research grant] Yeah, well, for us it's fraud directly. Because **you do it in order to win money**.	PMI
**INTENTIONAL**	

For me a bad scientist is someone who actually intentionally knows that what he or she is doing is wrong and might inflect the public opinion upon a publication. […] So for me, being a bad scientist **actually means intentionally being bad**. You can perform a research without being aware [of the rules] [...] That doesn't make you a bad scientist or a bad researcher.	RIO
If someone warned you that this is not okay, that it is bad science, and **you still continue**, then, yeah, it's also not okay.	RIO
There is big misconduct or minor misconduct, if we can put it like that. It's like when you **consciously know** you are really doing some change in your results to make them look beautiful and then get this publication in nature, for whatever reason, or when you are just tweaking here and there and the supervisors is telling you everybody does that, so you are able to do that [laughs]. There's **different degrees of seriousness** also in here.	PMI
Well if it's **willingly** then it's a… It's a border you don't cross.	PMI
**MORAL MISMATCH**	
[Sometimes researchers say] 'yeah but it didn't change the main results of my article, so what's the problem?' [...] OK **if the results are being the same, that's not the issue actually**, it should be the process also. And at that point you see that there is this **moral mismatch**.	RIO

Note: Bold added for emphasis

6. QUOTES FOR THE PROBLEMS OF SCIENCE

Select quotes to illustrate each findings on the problems of science.

actor

ISSUES RELATED TO THE PERSONALITY AND ATTITUDES

HYPER-AMBITION AND CULTURE

Personal egos and misplaced morals, or the 'bad apple' idea, was recurrently mentioned as a possible explanation for misconduct.

Quote	actor
"Internal it's idol-ness, the personal 'I want to be a big researcher', you always should be modest as a researcher I think. So the lack of modesty is the internal factor that makes people just improving their data a little bit., just adding something here and there, deleting something here and there…"	RIL
"I think that there is the egos, and the egos in science is still underestimated I think. yeah... [...] if you look at it when professors are doing it [i.e., committing misconduct], then it has a lot more to do with status, image, ego, trying to score, get off easy, yeah... I see that a lot more than it is of the pressure issue. [...] it's about image and scoring. "	RIO
"But the ones that really matter, that make it to the press, those are very often the leading universities and I think there the perverse incentives are big egos, big prizes, top publications... There, you know, if you manipulate your data, you become a big hero. And I think that's at the level of the promoter, and that's probably the biggest problem of them all. "	RIL
"You know that when you start fabricating papers to have like two papers a year in Nature, you don't do this for policy reasons. [...] You do this because you want to be kind of a king or a god in your discipline, and that's… well maybe narcistic, maybe psychopathic type of behaviors. "	PMI

Although they believed that passion, ambition, and tenacity were essential for success, interviewees said that, in excess, such aptitudes could lead to integrity failures

Quote	actor
"That's very important, because we're always talking about misconduct as if it's deliberate, as if you're cheating, I think maybe the most dangerous thing in research is in your wishful thinking, of self-fulling prophecies, you want it so badly that you will see it, you will see it the results, if you're out in a complex…"	FA
"And the good researchers, like I said, they're really passionate, they only think about their own research, they want to get things done, they want to get their results, so… What we usually see is that people then don't really follow the rules as they should, so they don't see why these rules are important. […] It's always that this researcher is really heart to… motivated to get the results done, and then bypasses procedures and rules, and that they don't see why these rules are in place and why they're so important to have them, why it's also protecting other people in the field… So this is mainly I think one of the reasons…"	RIL

Some respondents also associated attitudes and communication issues with cultural background and language barriers

Quote	actor
"…Sometimes I find that it's a matter of some cultural differences, in some cultures it seems that every meaning is justifiable to achieve the goal, and so they are trying to do anything they can do just to get their research published. So they will falsify data without a problem [laughs], they will not hesitate…" (EP)	EP
"I think [certain cultures] have this mentality that it's almost, you honor somebody by plagiarising them. And they just want to get their diploma so they can do a Post-Doc. in America." (RIL)	RIL
"I dare to say that [different cultures] have a slightly different opinion about rules. So they're more relaxed than [western Europeans] for example, and well... I think they didn't think it's that serious."	RIL
"He doesn't mind plagiarizing as long as he/she doesn't get caught. So yeah... That also had to do with his nationality."	RIL
"People have problem here with foreign students."	LT

"P1: Yeah, sometimes yeah, there are persons that you miss. And sometimes, I don't know, but sometimes we have the inspiration if we have to talk English but it's not the first language for the other person either...That's also sometimes a problem.<br>*P2: Yeah it's true, it's difficult sometimes!*<br>*P3: It's easier to explain something in Dutch. You give more details.*<br>*P1: Did they understand? Because you try, but you have the feeling, I don't know, after five minutes if they, if the effort's worth...*<br>*P2: And I feel also you're bonding better with PhD student from here eh? Because it's easier to talk. You just know them better.*<br>*P3: Small talk is easier much easier in Dutch and then of course you talk about everything then.*<br>*P1: Sometime it's also because we're not so good in English eh.*<br>*P2: Yeah we avoid it. and also, like, at lunch, we always speak Dutch. And then for them it's hard."*	LT

Social activities were thought to be helpful in creating a welcoming environment, but language barriers were said to keep the divide between Belgian researchers and international PhD students nonetheless

J: Sometime it's also because we're not so good in English eh.<br>*R: Yeah we avoid it. and also, like, at lunch, we always speak Dutch. And then for them it's hard.*<br>*E: Yeah, we try to learn.*<br>*E: I think that's a problem for some PhD students that they don't eat in the... They just do everything on their desk. A few years ago they ate at noon at the (cafeteria), where we all eat, but now all PhD students they eat at their desk and we don't get much contact anymore. It's a problem. IT's a communication problem.*	LT

ISSUES RELATED TO RESEARCH CLIMATES

LACK OF KNOWLEDGE OF GOOD PRACTICES

Some interviewees worried that insufficient training in integrity lead to a lack of understanding and awareness on these matters

"What I think is important though, is that, again in my generation, we were not made aware enough of that. A bit like gender bias; until you're explained what gender bias is, until you're explained what research integrity is, and what misconduct is, I think you will have more flexibility towards it because you just don't know where the red line is. Once you explain to people where the red line is, they will know when they cross it."	EP
"I don't believe that there are researchers who intentionally perform bad research, I do believe that there might be some researchers who are not aware of what the better practice or the best practice might be. And if they are informed about the better and the best practices in specific research and they follow these practices and they adjust their work methods, that's not a bad scientist."	RIO
"Now the more I look around, and to more we have our contacts with the universities, with the national administrations, and the research establishments, I realize that actually there are not that many courses. I realized that it is possible to go to a research position and never had a full course either in ethics or in research integrity."	PMI

INSUFFICIENT SUPPORT, MENTORSHIP, AND GUIDANCE

Students admitted that they lacked guidance, time, and support from their supervisors

"Well it was generally just my supervisor messing up. That was just the worst. Always the worst. Always. (laughs) And I'm not telling you… you know. So, not responding to emails, you know, for a very long time. Not being present. Not giving any useful feedback, if they give feedback, giving feedback that just makes your work worse instead of better… Not knowing how to supervise basically."	RCC
"I think everything I learnt, I learnt because of doing myself. I expected when I started my PhD project, that I would learn a lot from my supervisors, but now at the end of my PhD I think I didn't really learn a lot from them, so I'm a bit disappointed about that.".	PhD

This lack of support, in turn, often led students to feel lonely and frustrated, an element which was thought to be a red flag for integrity failure

"I get suspicious when PhD students are complaining, for instance, or feel alone or feel pressured to do things. Of course, in a certain sense this always happens. If you ask my PhD students, they will also say there are moments in which they were alone or pressured, so you cannot really prevent all of that but if that becomes too big, then I think there is something wrong"	RIN

Researchers also mentioned that they lacked support and guidance on how they should meet integrity and ethical requirements

Quote	Source
"Just having you as a researcher filling all these tick boxes, and not being responsible... [Funders] really should work on that. Also the same goes now for the data protection. They will make an extra box, and we should think that everything is arranged for data protection while no university in Flanders is ready for that by May 28th."	Res.
"If you look at the integrity and ethics part, that is something that is 'added' by few researchers. But it shouldn't be like that. It should be part of the research process, really completely connected in the research process for every researcher."	RIO

PRECARIOUSNESS OF RESEARCH CAREERS AND LACK OF SUPPORT FOR EARLY CAREER RESEARCHERS

The insecurity that early career scientists face about their future in academia was raised many times by participants who noted that early research careers are governed by short term contracts with low employment security

Quote	Source
"Yeah so for me it's because I'm in this end stage, the insecurity of the future is really something that I'm struggling with. Not every day, or not all day every day, but every day at least 5 minutes (laughs). […] The fact that you don't have a permanent position is also really ambiguous about it. I would like to have a little bit more future, and also not to have to find my own money all the time because I have the feeling that I'm not actually doing something myself. I'm constantly finding and looking for more money, so to hire people who are actually doing something."	PostDoc
"If I go for the academic world, I'm going to have to tell my wife, that was pregnant [at the time…], I have to tell my wife 'well we're going to a financially uncertain situation for at least 10 to 15 years. And maybe when I'm 30 or 35 and I have said no to you an enormous amount of times, I'm going to be so successful that I can say 'It's ok now, we can pay the bills.' But I'm still going to say no to you because I have to compete with the other people. Whereas if I choose another life or career, you get, for example a contract that lasts for your entire life, and you can build your life. You can start building your life. You can settle in a way, you can… You can make plans. Whereas in the academic world you can only make plans for 2 or 3 years. And that was the kind of life that I didn't want to <live>."	RCC
"If you really want to achieve trust and if you really want to achieve openness to mistakes, people should feel secure enough to do [be open and honest]. And I think one of the answers is 'you will not lose your job'. […] But the enormous discrepancy we have today between the job security of professors and the job non-security or whatever the English term is, of PhD students, post docs, and whatever, that is… That's not a good environment. […] …one of my colleagues for example, at every discussion he has with his promoter, [the promoter] always emphasised 'Well the funding is mine – because it was the funding of a project, not the funding of an individual – so I can change you. You're no vital… So the funding is mine, I can change the PhD researcher.'"	RCC
"I think, 90% of those who start with their research career after the master, I think 90% of them want to stay in academia. […] There's a target said by OECD for the number of PhDs each country should have. And we're still not there. Flanders, Belgium is still not there at the target. […] We're still under yeah... So we still need more PhDs according to OECD. Also here in this house we started to question this. Is this really so? Because we're now at a point 80 to 90% of those researchers have to find a job elsewhere, out of academia. […] They find jobs easily, that's not a problem, but not a job as a PhD the first years. They will work as a Master."	PMI

Several participants also noted that early career researchers are disadvantaged in funding opportunities

Quote	Source
"There's a certain starters package that I got, but it's not enough, you have to find your own money which is very difficult because you don't have the publication list. […] Because the first thing [funders] do is, they look at who is asking, and then at your resume and then they say oh no too junior or not well enough established in the field or, you know, stuff like that. […] you need money to publish, you need to publish to get money, you know, it's a circle."	RCC

Despite these issues and despite realising that the majority of PhD students will not continue in academia, there was a general feeling that leaving academia was a personal failure, not a failure of the system

Quote	Source
"I am the idiot that gave up [a professorship]. That's what it is, I worked my entire career to get at that point, I was in it for [a few] years and I gave it up. And so many people in the academy want to be in that position, and I gave it up. What kind of an idiot am I? […later in the discussion…] In the end I was like […] What's the chance that I'll ever help any patients, because that's basically why we all start doing it, to make a difference. But that's for the happy few, and those happy few have big names behind them and get money. They are not struggling to be at home, to put children to bed or whatever. The daily things that were too hard for me, and now that I don't have to do it anymore I'm a happier person. So maybe I'm not a real, real scientist."	RCC

"…people think when you do a PhD then the first and foremost career path is the one in academia. This starts changing, but there are all the phantom pains attached to it. People think that it's a kind of, a lots battle, a defeat, when they leave university and go to work in a company, or go to work for another agency."	PMI

INEFFICIENT CONTROL AND PERVERSE INCENTIVES

Interviewees worried that misconduct and detrimental practices often go unnoticed or unsanctioned

"People don't see the severity of [misbehaviors like gift authorship]. So then the gain is much bigger than the risk."	*RIL*
"While big industrial laboratories have standard operating procedures that are very expensive and standardised when it's not easy, in research institutions, we are missing them. [...] Sometimes this is missing. Right? So that can lead to sloppy research. "	*PMI*

The perverse impact of expectations of exceptional results was also noted, with respondents believing that these expectations discouraged researchers from being careful and from disclosing complete results

"You move by small steps in science as well as by big leaps, but the problem we have is that at the moment it's only the big leaps that are being rewarded. So people are incentivised to find these big bold claims, and you know, if you do an experiment with a small number… You know if you pick some situations, if you work in biomedical research with systems that are very noisy, like, you know, animal research of something, if you actually do experiment with small numbers of animals, you're going to have a much larger effect. This is statistically known, and it's not going to last the test of time. But why would you go through the effort of really verifying your claims until this effect becomes very small at which point you can't publish it anymore because you really wanted the big effect to publish in the first place… These are the kinds of perverse incentives that we have in the system at the moment."	EP

Short term financing schemes were also thought to increases the pressure for output and to intensify the perverse incentives already in place

"I think a researcher should not have these short-term financing situations. I think that's probably the worst perverse incentive you can give a scientist. I think you should have a tenure track where you require that a scientist proves him or herself, but once you have an established scientist, they should have some sort of basic funding which could be adjusted based on how they perform, but it should not be this 'yes/no' thing on a four year term which is what most grants are. Because I really need to deliver in four years, and that gives me perverse incentives."	RIL

CONSERVATISM

Interviewees considered that high risk research was important for scientific advancement, but they worried the reliance on established experts for reviews decreased the chances of obtaining funding for high risk or highly innovative research

"…peer review has this tendency to be a little bit conservative. Because since you have experts in your panels, people who already have proven themselves […] and also mostly are senior people, they can also sometimes, not all of them because you shouldn't generalize, but sometimes they can get a little bit conservative. Because they think that they have found the holy grail."	FA
"But also what we hear is that more and more, institutions, universities don't want to fund high risk research. So they only want to fund research that gives good results that can be used for society and so on. And if this would really be the case, in my opinion that's a very bad direction we're going into. Because research, especially fundamental research, should be able to lead to negative results."	PMI
"This is my main issue with the situation today. That there is so little opportunities to start something new where you don't have any funding, where you don't have any room to do this. If you look at the funding agencies at Flemish level, also at European level, they always look at your track record, and they, and you should always provide preliminary data, and this is something that's really lacking. Someone with a brilliant idea with no funding will never be able to start this new idea. And this is for young researcher, but also for established researchers. Everyone is sticking to the system because the system is like it is. And this is for me a problem because there is very little room for new things. […] There are some... like in Holland you have for example pilot grants and some, but even then you need to show that you have every technology in hand to do this new idea. And this is really a problem for me. I think that many researchers are now playing at the safe side because they already have shown that they work in this field, they will continue in this field, and they will not go broader, because probably they will not get funding because it's a new idea and they don't have any evidence at work. And this is a system like it is. And you need these things otherwise you will not get funding. This is for me really a problem."	Res.

Some interviewees discussed that they knew of researchers who asked funding for low-risk ongoing projects in order to use the funds for high-risk innovative ideas. Although most disagreed with this strategy, the lack of an alternative and the conservatism of the current system elicited great frustration

"I knew a lab and a professor that's just asking every year on the same project, but the title of the project changed a bit, and it's already proven but they just submit it every year and every time they have funding. It's so stupid, every time they get funding for something that's already proven, and they just explain it and they turn it, they describe it in such a way that it's new, and then they get funding. So it's also really stupid that a lot of people that can have good research, or a lab that can have good research, it's not getting funding, and that there is people that already proved it and they can get funding. That's also an issue."	PhD
Participant 1: "But then it seems it's fraud, because you got funding and then you use maybe for something else. That's what happens to be creative."<br>*Participant 2: "Yeah or you already write a project where you already have the data and... so you're asking money for something that you already have done."*	Res

OVERSPECIALISATION, WORKING IN A VACUUM, AND LACK OF TIME FOR RESEARCH

Research work was described as highly individual, with researchers working in a vacuum, distanced from one another, potentially duplicating each other's efforts

"But there is still too much going on at the personal level. Everyone has his or her PhD Project. And in that case you don't work together that much, plus there are so many project that nobody has a clue anymore what is going on in the institute."	RCC
"I'm sure there's an enormous amount of research that's just being duplicated over and over again, where we put in money that has no use because it is already known, we do not publish about it, and there are so many researchers, so it's very difficult to like interact with your researchers to know... Very often our researchers don't even know what is happening within their own buildings, so "Oh you're doing that?" So you know all these relations are completely missed. And that, I think is a waste of money, obviously[…] It's a very big consequence of the fact that that there are a lot of researchers and that they are everywhere around the world and that they have, that they are in different disciplines and different branches, so that there's a lot of barriers in research, that create, yeah... issues."	RIO
"In Dutch we say ZAP, zelfstandig academisch personeel [independent academic staff]. So they're academic staff, but independent. Independent academic staff. They're individuals who chose to sometimes work together. That's it. It's an individualistic group that, because of the necessity of their work, sometimes collaborate. But if they can, they will not…"	RCC

Collaboration was seen as important, but as something that researchers were sometimes reticent to undertake

"[… In discussing with my supervisor] I said 'Ah maybe it's interesting to collaborate with this person because his field of expertise is in that' and [my supervisor] was like 'No, we're going to try first on our own.' And I was a little bit disappointed with this reaction because I thought the other researcher was always saying like 'Ah you're also doing the things that I actually also would like to do' and like, it was perfect to get to work together on it. But my promoter said No. Maybe if we really need him for the knowledge or the content then... So I was a little bit disappointed about that, but, yeah..."	PhD
"[Researchers] always say [collaborations are] important and you have to do it, and we are doing it… But they're still not doing it enough because when it comes down to the discussion of who will be the first of the last author of a paper, then they completely forget collaboration and they would put their names first."	RCC
"I think it's also a problem that no one wants to share their unpublished data because they are scared that someone else will go and take the data and will publish first and then, you don't have it anymore."	PhD
"My promotor is always so reluctant to let me go and show the data <to> other people. Because we're a small center, it's the first time that we work with a hospital, and it's the first time that we do work with [specialised] physicians so it was very difficult to get it moving […] Sometimes I do understand, but sometimes I'm also like, I don't really like this kind of environment, it struggles with my personality a lot."	PhD

The lack of consideration for personal skills and the inflexibility around the pillars of research, teaching, and services further raised frustration and were believed to disregards personal skills and to discourage team efforts

"The advantage of academics is that you have many tasks, but this is also a disadvantage. Sometimes you have to do everything, you have to be good <in> English and <grammar>, in statistics, in everything, and... which is not always our expertise and also neither our interest."	Res.

"The problem is that today we cannot deliberate between those three pillars. And I believe that if you're excellent in education and you spend 80% of your time in education and you do only 20% in research, and you don't get your criteria for research but you overdo your criteria in education, why not make a balance?"	RIL
"I believe there are very good researchers that have to teach and that suck at it, and I believe that there are good teachers that have to do research and suck at it. Or at least are not top notch at it. But no we all have to be equally good at both and we all have to divide our time exactly the same."	RCC

PRESSURES

Pressures imposed on researchers and the culture of publish or perish were the issues that were mentioned by the biggest number of interviewees

"The pressure is huge. You know, basically your career depends on it. And so when you are in a system in which basically your next paycheck, your next grant will be dependent on the results, I've never thought about it, but I can imagine that you will have some people think 'Well… Why don't just, you know, make it up?'. "	PE
"…the more mundane reason I think has a lot to do with time and publication pressure and the pressure for funds, fundraising and things like that. Which puts an enormous pressure on people to produce results, to publish results…"	RF

The key used to distribute structural funding between institutions was particularly discussed for being at the onset of the publish or perish culture

"The reason therefore is very clear. The reason that publications are so paramount in the assessments, in scientific assessments is that in this country and also in other countries, the way universities are funded and get their structural research funding is calculated mainly on the basis of the number of publications their staff has produced."	FA

Yet, the real problem was said to reside in the implementation of the distribution key at the individual level within institutions

"The BOF key was actually only created to divide the money under the universities. And what we see is that the same parameters are being used within the universities themselves to fund the individual researchers. That was never our intention… So that's the negative effect of this key that we never wanted."	PMI
"The BOF key is just one thing. It's a distribution rule that has to divide a pot of money among five universities. In one way or another, you will always need some distribution mechanism. The BOF Key – That's also why we never report on individual researchers – but what the BOF key does is just aggregated at the level of a university: count PhD output, count publication output (certain type of publication output I'll come back to that). […] I know from hearing and feedback that I get that certain institutions try to, what I would call extrapolate, or interpolate the BOF Key into individual level research output. I think that's wrong, that's even stupid. But it may happen. But the BOF key it's not there to do this."	PMI

Worries around the pressures imposed on researchers were predominant, but interviewees also revealed that pressures are multifaceted and that they also affect different actors

"…to even conserve [their] share, make sure that [they] will not get less than the previous year, [universities] have to work always harder, [they] have to produce more publications. Because if [their] competitors – other universities – produces more than [them], then the share of that same amount of money will decrease. So this is the so called rat race, there is a lot of criticism about it, I can understand that criticism, because it also puts a big pressure on us, because people have this idea, this feeling that the institution is pressing them to publish always more. And this publishing can also only be done of course on the base of new research actions, also the pressure on our organisation is increasing. And, well I don't think I'm saying revolutionary things here, this is not a very sound environment to work in. So I think that this should be reassessed."	FA
"… there isn't anything that we can do. For a very long time we were frustrated because we didn't have an impact factor. Now we do. It's only… I would call it a trace impact factor, it's very, very low. And I think that you might be idealistic and make presentations at […] conferences about how we should get away from the impact factor. But if the ministry of education says that's it, we are using the impact factor, or the ministry's index is based on the index factor, who am I to… it's very idealistic to say let's have another, let's ignore impact factor. You can't ignore impact factor, so we can't do anything, we haven't really figured out how to make our impact factor higher. Also it's very frustrating that the impact factor is higher in certain disciplines than in others, so in my discipline, I don't know probably the leading journal I think is one point something, the leading one. So how can we go any higher, yeah..."	EP
"We have to remember that we can only publish [a certain number] of papers per year, and we get sent [many times more than this]. So this is the sort of choice we have to make."	EP

Culture of profit and intolerance for failures and mistakes

The focus on outcomes and profit was said to forget the care that should be put in place to support and nurture good researchers

"In research you're not making research results, you're making good researchers. And you have to develop and support the people, and not just the research. And I think that that entire culture of care is missing too much. We see them too much as producers of research results, instead of 'we are making a good researcher that will, hopefully go on a lifetime making good research results'."	RIO

Interviewees saw the value and importance of mistakes in science and believed that researchers should be encouraged to disclose mistakes and negative results

"I think we should reward failure, rather than punishing it. Because those are the lessons that will make you do a better experiment, better design, etc. […] we have created a system where our excellence is geared towards success, and our impact is geared towards success […] So there is something wrong with what we are asking, or the way that we are asking it. And if we do not tolerate failure, and use failure as a motor to drive you to success, you foster misconduct and sloppy research."	PMI
"Of course if you work in healthcare you talk about human life, so it's understandable that you don't want to talk about your mistakes. In academia, that's not understandable. I don't understand why… Well of course you can understand this. But it's not justifiable. It's understandable, but it's not justifiable. Because if there is… If one place in our world should be a place where people are free to make mistakes, even though we pay them a lot, and we hope they don't make mistakes, then it's that place."	RCC
"And if we do not tolerate failure, and use failure as a motor to drive you to success, you foster misconduct and sloppy research. Most of my researchers would be very upset, but that's how it is! "	PMI
"So even research which does not attain the results expected, but which explains why it did not happen, is successful! Of course, it demonstrates something else. A new something, for example."	FA

The discomfort and fear from having to disclose mistakes however, was still vivid among interviewees.

"I think what is also a problem is the fact that it's still a 'taboo' I would say, just to come open with the fact within research "I made a mistake" in the past. We had something in the past in our group that there was… suddenly there was… everybody thought that a measurement was wrong. Something in the system and all of the data that were captured were therefore wrong, and <these> were already data which were published. And then it should be decided what to do. Should we do a correction to the journal or not? And there was a lot of pressure from the professors, because it came higher and higher in the university, and some people were afraid, and some were like 'Whatever!', and everybody had another opinion, the PhD students just had to follow... But I feel the big difference between some people who were very ethically committed, like we have to correct it and we have to send it to the journal, and others were like 'nobody will see it, it's in the past', and... Yeah, I saw a lot of things which should not have been happening."	Res.
"I never said to [my supervisor] that I made a mistake, never. I always said [to my supervisor] well I have these questions, we might want to do this, but I never said I did something wrong. No never. Because even with my promoter I didn't feel secure to be open and transparent about mistakes. And luckily, I didn't make mistakes that made sure that the research didn't work. So it was always mentioned. But I wasn't transparent about mistakes. Never was in academia."	RCC

Many also admitted that negative results are often abandoned before publication, and that instead, experiments are repeated or reorganised to focus on positive results

Interviewer: "Does it happen that you see an experiment that you've taken part in, and that, it was negative and then it's just dropped? "<br>Participant 1: "All the time!"<br>Participant 2: "A lot!"<br>All together: "YES!"	LT
"But that's really difficult, if you have a promoter who is pushing you really hard, for example there are some people here that have to repeat experiments let's say for 5, 6 times to get positive data out of it, and then it's just one time that the data is positive and that's good and all the other five times that the data was negative they just ignored..."	PhD
"And we know after years [PhD students] have to repeat, repeat and when there's something positive, it's a surprise to them."	LT

"The result has to be significant.... And also if you have – also that was a big problem – that you have the results, and then you're looking, you're like 'data fishing' eh? You're looking for... somewhere there will be a significant result then you can ignore all the rest. And that it's a little bit solved with clinical trial registrations, but not a lot because your data analysis plan is not detailed. And then sometimes I think everybody is like 'Are we going to sell the story positive or negative?' That's the first thing. And if it's negative you will find a way how to do it and if it's positive you will also find a way to do it."	Res.

Researchers supported that the unwillingness of journals to publish negative results and the expectations of funders were at the source of the problem

[Discusses an example where negative results on virus research were published in a journal together with an open discussion of scientists.] "At the end it seemed like in certain instances this virus was really important while in other subpopulations of patients it didn't really matter. So, but... in most other cases, you really have to fight to get your paper out there if you are behind this one big story. And it's more difficult to publish while you don't see if your results are positive."	Res.
"I had a colleague that I think it took them five or six years to get it published because it was negative results, but very interesting study, also it was never done before. And it took them six years to finally publish and then it was in PLOS One, and they almost accept everything, but it was very difficult to get it out there. And I would also lose motivation, I think eventually. But it's good that they kept trying and eventually published it."	PhD
"Often also the colleagues here were talking about experiments that don't work, often you have experiments or studies that do work, but when the results are for example that an intervention doesn't work or something… I think that's a very valuable insight too, because then you can also say to the clinical field, well you don't have to try that because we studied it and it doesn't work. But a result like that is not sexy enough to publish. So often you submit your paper, and you're four journals further when you get an acceptance..."	PhD
"But I think that's also again the fault of science a bit, that they really want positive publications. And if you can also publish the negative results, which is also important, and if you really did a lot, and you really did a good job, and you really can have your PhD but you don't have any positive results, it's also stupid because you can't publish them. Because most of the time they will be rejected."	PhD

Yet, editors or publishers and funders mentioned that they would be happy to consider negative results, but rarely receive them from researchers

"We have a company doing the review process by external experts, independent expert, who judge if there are deviations in comparison with what was originally planned. If these deviations are well justified, well tackled, and voila. Then the project is continued."	FA
"We're trying to do things about that. We have created journals where you can publish anything, you can even publish negative results. Better, one of our publishers was speaking with editors in community [a specific topic]. And there was a complaint about the fact that there was no outlet for negative results, and they came to the conclusion 'Why don't we create a journal? […] They created a journal dedicated to negative results in [this specific field of research]. Two years later, they closed it. No submission, whatsoever. No submission! Why? I don't know."	EP
Participant 1: "We usually don't receive any such papers [laughs], but if you will find data useful, even though the results are negative we will publish such papers, but usually we don't receive them at all, I'm not sure why [laughs].<br>*Participant 2: I don't remember we received papers negative results, but we would publish it, we would also publish a paper that repeats another study, to… and confirms the results, for yeah, for reproducibility purposes, that's very useful to have this, this backup, yes."*	EP
[RIL who also acted as EP] "If you would know that there was already somebody who tested your hypothesis with negative you would stop doing that. So the negative results are for me the most positive publication, but since the five years that I am an editor I have never seen one negative result."	RIL

UNREALISTIC EXPECTATIONS

As discussed in the perverse incentives, expectations that researchers should produce extraordinary findings were believed to encourage researchers to embellish their findings

"And in [our journal], you know we would always ask first of all, is this needed, is this a really big step forward, is this clinically important, is it novel enough, you know…"	EP
"Researchers are incentivized to really get something that is extraordinary, and ground-breaking. And let's face it, all the research in biomedical research is not ground-breaking and extraordinary. Most of it is not."	EP

"To really get a high paper, you should really be good in storytelling. And this doesn't always say a lot about the real science behind it. So the 'commercial' part or should I say 'communication' part of that sometimes leaves good scientists in the cold because they're not... they really have nice data but they're not good enough to sell it. This is something that sometimes is frustrating to me..."	Res.
"But I'm also – that's also a kind <of> problem – I'm not like, I won't exaggerate what I can do. I'm what you see is what you get, everywhere. So it's very difficult for me to write grants, because you have to exaggerate and bluff and I hate doing that, because I'm… What if I can't do it? And that's something that you should not even think about probably, to be able to write a good grant you have to exaggerate, and you have to believe you will be able to but it's… I'm too practical for that, so it takes me a lot of time to write stuff like that."	RCC
"If we start reading, all of us, in all the national system, all the impact sections of the proposals, in principle we shouldn't have any problems in this world. Because the impact promises that we will solve it. And we know that's not true. So there is something wrong with what we are asking, or the way that we are asking it."	PMI

Interviewees also revealed that researchers were expected to devote themselves to science without the desire for personal benefit

"I am quite amazed when I listen to some discussions that are triggered by PhDs students and Post Doc, where they consider their PhD work to be kind of a job. This is not a job. This is an opportunity to pursue a scientific interest. I would never call 'doing science' a job." […] "…being passionate about science is almost like being an artist. You live in poverty because you want to pursue your art."	PMI
"If you want to raise the quality of a university you need people with commitment who do it for the honor if you like. Sure, if it's paid on top of that that's great, but you should have an intrinsic motivation and not this extrinsic motivation " […] "I think people have to realize when you do a PhD, it's a stressful thing, you really are going to get the highest degree there is at a university, it doesn't fit between 9 and 5."	RIL
"I'm somewhat older, but I have the impression that younger people have […] somewhat a different work/private balance than I had. And I think that people sometimes could put more energy in their work."	RIL

But several researchers who left academia criticized this perspective, stating that such expectations impeded on personal life and even impacted on psychological and emotional well-being

"Then you have to go abroad. You forget about your family and your children, you go abroad for two or three years or half a year, and you try to come back, get some more publications and maybe you get a professorship." […] "I think a real academic is someone who has such strong principles that he shouldn't be concerned about paying his loan, making sure he pays his rent, or whatever, her rent. He should live by his principles or her principles. That's the idea of a scientist. If you make up a real scientist then maybe it's someone who is not like us. Like a hero of some kind. […] But my experience in the scientific world is that scientists are much more like the other people. They're also concerned about paying the rent."	RCC
" I just don't want to have a high-pressure job like this anymore. I just don't want to do it!' Because it was a very… Like you have to travel around a lot, you have to interview all these [high ranked] people, you have to visit all the fairs… You know, in Macau, in Rio de Janeiro, in Ankara, and it sounds like a dream for many people. But I was just… I'm already tired just thinking about it. So I was so burnt out I was just like… I just don't want to have this kind of life."	RCC
"I was stressed out completely […] I went to the doctor, [I] was on antidepressants, [I] was in therapy…"	RCC
"I got a therapist and I worked through it with her and, you know she said… Like whenever she said 'Maybe you want to start thinking about [your work]?' I would just start to cry, so she was like OK, too early! […] Yeah. It was awful."	RCC

COMPETITION, HIERARCHY, AND ADVANTAGED DUE TO NETWORKING

Competition was thought to be necessary to drive progress, but its dominance was criticised for undermining openness and joint efforts

"Competition in the academic world is so strong, so fierce that in the end I experienced it as a… not a war, but a hostile environment." [later in the discussion…] "And it's not necessarily a bad thing, competition is not necessarily a bad thing, but sometimes… It's a good thing when it's a means to an end. But my feeling of the academic world is that most of the time it becomes an end instead of a mean. That's why you do it, because you want to be in the competition, you want to make sure that you're the real expert and not the other one etc. But fine."	RCC

Hierarchy was said to hinder researchers' openness and willingness to contradict more senior colleagues or to blow the whistle on bad practices

Interviewer: "Let's say you're talking... you're in a lab meeting, a student presents his data and then you know that something about the data... You've done the test and you know that he's reporting something wrong, not because he wants to. Then you say 'no no, that should be this..." and the PI says 'No, he's got it right.' Would you push?"
P1: No, I would not.
(All laugh, all 'No' except one case).
[…]
Interviewer: "I know that that's very theoretical, but why do you think you cannot go against your PI? Like what's your relationship? What's the reason that says that you're a technician and you're under a PI?"
P1: "I don't think that's the reason. I have a feeling that he's much smarter than us. Smarter than I am."
P2: "Yeah, the knowledge that we have is much smaller than theirs."
P3: "That depends on what you know. If it's pure technically..."
P1: "Yeah but I'm doing a technique which he excels so what can I say to him... I'm not nearly as good as he is..."

LT

[After asking participants if they would stand up against their supervisor for inadequate authorship practice (gift authorship), one did and explained the story, while others were uneasy about the idea]

P1: "I think I'm too afraid to do it at this moment. I'm not publishing yet, but at this moment I don't think I would say anything. I think I'm too scared, too afraid... I don't know... Too afraid that they will... not hate me, but... yeah..."
P2: "They will make it hard on you further on the project."
P3: "Yeah, I'm scared that they would make everything hard for me. And then... I don't think I would. Maybe in a couple of years away, but at this moment I think I would be too scared."

PhD

Many researcher and students provided examples in which networking could help them get ahead

"Once you are in the network, you can also rely upon them and say 'please do me a favour because I did a favour to you."

Res.

"…we were going for [a high impact journal], and then we were not writing the paper we were spending all our time trying to get the editor and the reviewers that they knew that the paper was coming so that once the paper was there all these people were involved and engaged and then it was either accepted or not. So it really depends on who you know and who you don't know. And that's why sometimes I start behaving like that… 'Oh I would like to have a paper in one year in that journal' and I start writing them and seeing them at conferences (laughs) 'Hi, yes I'm thinking about submitting a paper what do you think about the idea' and it really really helps. So it's really not as unbiased as you would like it to be." (Post-Doc)

PostDoc

But the fact that researchers may benefit from favours from their network was also criticized for threatening the fairness and objectivity of science

"I feel like I have less and less confidence in publishing with the fact that 'who is going to be the reviewer?' 'Is he biased?' 'Is it the journal?' It's like some politics that you.... I don't always believe that the best results are published in the best journals." (Researcher)

Res.

"…it's just the people that have the money that get the money. Because they're all in the commissions or they have co-workers or close collaborators that are in the commissions, and they just give each other money all the time."

RCC

Contradicting the perspective that networks could bias decisions, funders and editors or publishers maintained that the review processes in place avoided conflicting interest from researchers' networks

Interviewer: Some researchers mentioned that one of the ways to be successful is to make good networking with editors, because that opens the door to the journal. [all laugh], What is your view on that?
"P1: Wishful thinking [laughs]. […] We have such strict policy that I turn down my best friend when he sent a paper, so no networking here was really known [laughs], was not for… we have a same policy, so well sometimes perhaps if the paper is from someone you know and you are not really sure whether you are objective enough to, you know, to say okay it's good enough, or for our journal or not, but you are clearly depending on the reviewers to give you that, and you are sending it to independent objective readers outside of the community, so it's…
Interviewer: Is that the same for <everyone>?
P2: Yes, by reviewers, we also ask reviewers if there is any conflict of interest with the authors, and well I remember one case, one of our former editors and chief was really productive in submitting articles to the journal, but at least two of those were rejected! [all laugh] […] If one of the editors is author of a paper, we will keep him or her away from this review process.
P1: We also have this policy, but to be fair I must say that there are no journals in our country which are doing that by networking, I will publish your research, you will publish mine in your journal, and it's usually not in our field [laughs]. And it's not happening in our field or technical field, or life sciences, or medical, not sure about medical, I'm not tracking that. But those fields which I am usually are…
P3: It cannot happen."

EP

Quote	Participant
"The majority of our panels are foreign members. So… Or I should say because nationality is not relevant, but people with a non-Flemish affiliation. It can also be the other side of Belgium, but mostly people from other countries, even other continents. And a new regulation that is adopted recently, the chair of non-Flemish members is even higher than it is today. […] So you always need more expertise than is available in your own region. But of course, and that is also what we believe, and this is a, let's say general honored belief in other organisations in other countries, that not too many people should be too close on the applications presented here because always, not always, but very often there occurs types of conflicts of interest. And this is very normal, is can be positive conflicts of interest, so people wanting, on a, let's say willingly, or knowingly, or maybe a little bit more unconsciously helping people they respect, they hold in high esteem. But there's also of course negative conflict of interest people who are your competitors and you want to harm. So to avoid this, it's good to have people who are a little bit on a distance from the application. That's the idea."	FA

PUNITIVE, NOT PREVENTIVE

A few interviewees worried that the scientific culture focused on punishment rather than prevention, and that in doing so, it may waste opportunities to learn from misconduct

Quote	Participant
"I personally have a big issue because research is one of the few social activities where a second opportunity is rarely given. […] If someone did something terrible, of course they should be punished. […] Keep them in. Teach them how to do it correctly, and help them teach other students of what happened. That for me, would be a holistic approach. You send that person out, what will happen? You send them out of the university of Hasselt, he goes to University of Maastricht, and he gets accepted there probably in the same position. So, who won? There is no closure. The university of Maastricht doesn't have the smallest idea of who — well maybe the proximity they might call the professor eh, they might – but they can go somewhere else, they don't have to go across the border. Even sometimes if they go in the same country, people don't check. But you have not achieved anything. So for me, and I will try to have a discussion on that, but the scientific community itself is extremely reluctant to discuss it. Once I told colleagues, people that deal with misconduct at the national systems, that I would like to organize a training for commission officials and researchers by inviting people that had been found guilty of misconduct. He was not even entertained as a joke. He was really: "No way, that we will give them a forum". But you don't, you use them. […] I think it's better to focus our attention on prevention rather than on punishment. Right now most of our systems are for punishments, and much less on prevention. And I think we should have a policy shift there, if at all possible."	PMI
"In science of course, if you really retract a paper, that is really the final stage eh, of misconduct. If that communicates then, today it really gets to the press, things like that. But the scientist should also be able to say 'OK I made a mistake at that moment, how can I make it up again?' Because once you have a mistake of course you try to hide that, and then people build up on it."	RIL
"If there is [a suspicion of misconduct], everybody will say 'OK I don't want to...' That's contagious, I go away from this person who did maybe fraud. So, they are punished already just by being accused. I think this is something which is still not in order in our system"	RIL
